# N-acetylcysteine ameliorates oxidative stress-induced decline in sex hormone-binding globulin in HepG2 cells and polycystic ovary syndrome rats

**DOI:** 10.3389/fphar.2026.1930645

**Published:** 2026-10-13

**Authors:** Xinyuan Zhang, Qiuyi Wang, Suiyan Li, Wei Huang, Huai Bai, Qingqing Liu, Ping Fan

**Affiliations:** 1 Laboratory of Genetic Disease and Perinatal Medicine, Key Laboratory of Birth Defects and Related Diseases of Women and Children, Ministry of Education, West China Second University Hospital, Sichuan University, Chengdu, Sichuan, China; 2 School of Life Science and Engineering, Southwest Jiaotong University, Chengdu, Sichuan, China; 3 Department of Obstetrics and Gynecology, West China Second University Hospital, Sichuan University, Chengdu, Sichuan, China

**Keywords:** gene expression, HepG2 cell, N-acetylcysteine, oxidative stress, polycystic ovary syndrome, rat, sex hormone-binding globulin

## Abstract

**Background:**

Low sex hormone-binding globulin (SHBG) levels are a significant indicator of hyperandrogenism severity in patients with polycystic ovary syndrome (PCOS) and are associated with an increased risk of reproductive and metabolic diseases. Oxidative stress suppresses the production and secretion of SHBG. However, the effect of the antioxidant N-acetylcysteine (NAC) on hepatic SHBG expression remains unknown. This study investigated the effects of NAC on SHBG expression and the underlying mechanism in HepG2 cells and a rat model of PCOS.

**Methods:**

HepG2 cells were treated with testosterone propionate (TP), oxidized high-density lipoprotein (ox-HDL), oxidized low-density lipoprotein (ox-LDL), and NAC. Female Sprague-Dawley rats aged 28 days were randomly allocated to eight groups: control, PCOS, and control and PCOS groups treated with NAC at 100, 200, or 400 mg/kg body weight. The PCOS rat model, induced by TP and a high-fat, high-sugar diet, was treated with different doses of NAC for 8 weeks. The expression of SHBG, hepatocyte nuclear factor 4α (HNF4α), chicken ovalbumin upstream promoter-transcription factor 1 (COUP-TF1), and peroxisome proliferator-activated receptor-γ (PPAR-γ) was assessed in HepG2 cells and rat liver tissue. Metabolic, hormonal, and oxidative stress indices were analyzed in the rat model.

**Results:**

In HepG2 cells, TP and oxidized lipoprotein treatments significantly suppressed SHBG and HNF4α expression, while NAC treatment reversed the inhibitory effects induced by ox-HDL and ox-LDL (*P* < 0.05). In PCOS rats, NAC intervention increased the mRNA and protein levels of SHBG by upregulating HNF4α and downregulating COUP-TF1 and PPAR-γ expression in the liver; increased serum SHBG; reduced hepatocytic reactive oxygen species levels (*P* < 0.05); and ameliorated obesity, estrous cycle abnormalities, hepatic steatosis, circulating sex hormone levels, and the lipid profile, with 200 mg/kg NAC showing the most favorable overall pattern among the three doses tested.

**Conclusion:**

NAC treatment ameliorates oxidative stress-induced decreases in SHBG and HNF4α expression in HepG2 cells and may restore hepatocytic SHBG production by upregulating HNF4α and downregulating COUP-TF1/PPAR-γ expression in rats with PCOS.

## 1 Introduction

Polycystic ovary syndrome (PCOS), recently renamed polyendocrine metabolic ovarian syndrome (PMOS), is a prevalent disorder that affects approximately 10%–13% of women of reproductive age worldwide. The syndrome is characterized by ovulatory dysfunction, hyperandrogenism (HA), and polycystic ovarian morphology, and is often accompanied by overweight/obesity, insulin resistance (IR), multiple metabolic disorders, reproductive dysfunction, chronic low-grade inflammation, oxidative stress imbalance, and increased risks of type 2 diabetes (T2D) and cardiovascular disease (CVD), thereby threatening women’s long-term health (Teede et al., 2026; Tay et al., 2024; Teede et al., 2023; Mancini et al., 2021; Escobar-Morreale, 2018). Although the occurrence of PCOS is believed to result from an interaction between genetic and environmental factors (Moolhuijsen et al., 2026; Zhao et al., 2025; Filippou and Homburg, 2017), its exact etiology and pathogenesis remain poorly understood.

HA, which clinically manifests as hirsutism, acne, and alopecia, and biochemically as elevated androgen levels, is a core diagnostic feature according to the Rotterdam criteria and is a key driver of PCOS pathophysiology (Wang et al., 2023; Teede et al., 2023). In addition to its reproductive consequences, HA is intrinsically linked to abdominal obesity, IR, dyslipidemia, increased oxidative stress, T2D, and CVD (Wang et al., 2023; Zhang et al., 2017; Rosenfield and Ehrmann, 2016).

Sex hormone-binding globulin (SHBG), a glycoprotein predominantly synthesized and secreted by hepatocytes, regulates androgen bioavailability through its high-affinity binding to circulating sex steroids, with the following binding affinities: dihydrotestosterone > testosterone (T) > estradiol (Aydin and Winters, 2025; Zhu et al., 2019). Sex hormones bound by SHBG have no biological activity; only 1%–2% of unbound androgens can access target tissues to exert biological effects under physiological conditions (Aydin and Winters, 2025). Low SHBG levels increase bioactive androgen concentrations and are significant indicators of HA severity in patients with PCOS (Szybiak-Skora et al., 2025; Sun et al., 2021; Zhu et al., 2019). Additionally, low SHBG levels are associated with an increased risk of obesity, IR, PCOS, T2D, metabolic syndrome, gestational diabetes mellitus, CVD, female and male infertility, and certain cancers (Szybiak-Skora et al., 2025; Aydin and Winters, 2025; Qu and Donnelly, 2020). A recent study indicated that women with PCOS and high SHBG levels have relatively mild metabolic and neuroendocrine disorders, favorable reproductive outcomes, and the lowest incidence of diabetes and hypertension (Gao et al., 2025), further establishing the importance of elevating SHBG levels for the prevention, treatment, and prognosis of PCOS.

The *SHBG* gene is located on chromosome 17p13.1, and contains eight exons, with a proximal promoter region containing two cis-elements, FP1 and FP3, in hepatocytes (Szybiak-Skora et al., 2025). Hepatocyte nuclear factor 4α (HNF4α) binds to the FP1 and FP3 regions and estrogen receptor α (ERα) binds to the FP3 site to promote *SHBG* transcription, whereas peroxisome proliferator-activated receptor-γ (PPAR-γ) and chicken ovalbumin upstream promoter-transcription factor 1 (COUP-TF1) can compete with HNF4α for binding to FP3 and FP1, respectively, and repress *SHBG* transcription (Szybiak-Skora et al., 2025; Zhu et al., 2019). Additionally, thyroid hormones, adiponectin, obesity, weight loss, IR, tumor necrosis factor α, and interleukin-1β can indirectly regulate *SHBG* transcription by affecting the levels of the transcription factors HNF4α, PPAR-γ, and COUP-TF1 (Szybiak-Skora et al., 2025; Aydin and Winters, 2025; Zhu et al., 2019). Expression of HNF4α and SHBG in the liver is decreased in rats with PCOS and non-alcoholic fatty liver disease (Hu et al., 2024). Pioglitazone, metformin, and/or exenatide treatment upregulate the protein levels of HNF4α and SHBG in liver tissue and increase serum SHBG levels in rats with PCOS and IR (Zhao et al., 2023; Xing et al., 2021).

Oxidative stress is a well-established pathogenic factor in PCOS (Sun et al., 2021; Gonzalez et al., 2019; Zhang et al., 2017; Murri et al., 2013). Studies have found that patients with PCOS and HA exhibit more severe oxidative stress and impaired antioxidant properties of high-density lipoprotein (HDL) compared with their non-HA counterparts (Zhang et al., 2017; Zhang et al., 2015)**.** Oxidative stress represses the production and secretion of SHBG by downregulating HNF4α in HepG2 cells and may be a significant factor promoting the occurrence of HA in PCOS (Sun et al., 2021). N-acetylcysteine (NAC), an acetylated form of L-cysteine, is an antioxidant because of the reducing activity of its thiol group and its precursory role in the formation of glutathione (GSH), an important natural intracellular antioxidant (Liu et al., 2023; Devrim-Lanpir et al., 2021). As a safe, effective, and economical nutritional supplement, NAC has been widely used to treat oxidative stress-related diseases in humans (Vina et al., 2025; Fang et al., 2024; Shahveghar Asl et al., 2023; Liu et al., 2023; Devrim-Lanpir et al., 2021). NAC has been shown to increase insulin sensitivity, progesterone levels, and endometrial thickness, as well as improve androgen levels, pregnancy and ovulation rates, and metabolic abnormalities in women with PCOS (Vina et al., 2025; Shahveghar Asl et al., 2023; Liu et al., 2023; Sandhu et al., 2021; Devi et al., 2020; Thakker et al., 2015). NAC supplementation also significantly improves estrous cycles, endocrine-metabolic parameters, and ovarian oxidative stress in mice with PCOS (Fang et al., 2024) and differentially attenuates placental and uterine ferroptosis in a pregnant PCOS-like rat model (Hu et al., 2021). However, the effect of NAC on hepatic SHBG expression remains unknown. HA has also been shown to decrease SHBG expression in PCOS; however, its molecular mechanism remains unclear. Therefore, in this study, we investigated the effects of NAC on SHBG expression and its underlying mechanism in HepG2 cells and in a PCOS rat model induced by testosterone propionate (TP) and a high-fat, high-sugar diet.

## 2 Materials and methods

### 2.1 Lipoprotein isolation, purification, and oxidation

Low-density lipoprotein (LDL) and HDL were isolated from the sera of volunteers using sequential density gradient ultracentrifugation (Sun et al., 2021; Zhang et al., 2006). Oxidized LDL (ox-LDL) and oxidized HDL (ox-HDL) were generated from native lipoproteins via Cu^2+^-induced oxidation, as described previously (Sun et al., 2021; Gerry et al., 2008). The protein concentration was determined using a modified Lowry Protein Assay Kit (Pierce Biotechnology, Inc., Rockford, IL, USA). The oxidation indices (conjugated diene and total oxidant status) of the lipoprotein samples were quantified colorimetrically as described previously (Sun et al., 2021; Zhang et al., 2017; Gerry et al., 2008), and the results are shown in Table 1.

**TABLE 1:** Levels of conjugated dienes and total oxidant status in lipoproteins and oxidized lipoproteins.

Lipoproteins	Conjugated dienes (μmol/g protein)	Total oxidant status (μmol H_2_O_2_ Equiv./g protein)
HDL	127.64 ± 33.94	4.72 ± 2.00
ox-HDL	179.74 ± 7.81	9.25 ± 1.38
LDL	109.86 ± 43.26	5.07 ± 1.20
ox-LDL	281.15 ± 32.57	39.20 ± 15.64

Values are presented as the mean ± standard deviation (n = 4 in each group).

HDL, high-density lipoprotein; LDL, low-density lipoprotein; ox-HDL, oxidized HDL; ox-LDL, oxidized LDL.

### 2.2 Cell culture and treatments

The human hepatocarcinoma HepG2 cell line used in this study was obtained from the tumor cell bank of the Chinese Academy of Medical Sciences. HepG2 cells were cultured in Dulbecco’s modified Eagle’s medium (DMEM, GIBCO, Life Technologies Corporation, Grand Island, NY) supplemented with 10% fetal bovine serum, 100 U/mL penicillin, and 100 μg/mL streptomycin. The cells were seeded in 6-well plates, and the medium was replaced with serum-free DMEM at approximately 70%–80% confluence for a 24-h serum-starvation period before experimental treatments.

Based on reported NAC concentrations used in HepG2 cell experiments (Yedjou and Tchounwou, 2007; Sakasai-Sakai et al., 2020), we selected NAC concentrations of 0, 1, 5, and 10 mM to treat HepG2 cells for 48 h in the pilot experiment. HepG2 cells treated with 1 mM and 5 mM NAC had morphology similar to that of control cells, whereas 10 mM NAC caused noticeable morphological alterations and cell detachment on inverted microscopy. Therefore, based on the pilot experiment and previous reports, 5 mM NAC was selected for subsequent experiments.

TP (Sigma-Aldrich, St. Louis, MO, USA) and NAC (Sigma-Aldrich) were dissolved in anhydrous ethanol and phosphate-buffered saline (PBS), respectively. The medium was then replaced with serum-free medium containing TP (5 nM), NAC (5 mM), lipoproteins or oxidized lipoproteins (50 μg protein/mL), 10% sucrose-PBS, or the corresponding solvent controls, and the cells were incubated for 48 h. Throughout the subsequent experiments, cells in all treatment groups were routinely monitored using inverted microscopy, and no signs of cytotoxicity, such as shrinkage, floating, or detachment, were observed.

### 2.3 SHBG quantification in the culture supernatant

Following 48 h of treatment, SHBG concentrations in HepG2 cell supernatants were quantified using commercial enzyme-linked immunosorbent assay (ELISA) kits (E-EL-H6238, Elabsciences Biotechnology Co., Ltd., Wuhan, China) according to the manufacturer’s protocol. The relative levels of SHBG in the culture supernatant were normalized to total cellular protein.

### 2.4 Establishment and intervention of the PCOS rat model

All animal experiments were approved by the Experimental Animal Management and Ethics Committee of West China Second University Hospital, Sichuan University (approval number: 2023-003). All animal procedures complied with the guidelines of the Animal Care and Use Committee of Sichuan University.

Sample size calculation for animal experiments: In the absence of background data on the effects of NAC on SHBG in PCOS rats, the sample size was calculated based on the effect of pioglitazone on serum SHBG in PCOS rats in a previous study (Zhao et al., 2023). Based on SHBG levels of 89.25 ± 3.01 nmol/L in the untreated PCOS group (n = 6) and 100.7 ± 5.69 nmol/L in the pioglitazone treated PCOS group (n = 6), the common standard deviation is 4.55. Considering a two-tailed significance level α = 0.05 and target power of 0.8, an online Power and Sample Size Calculator indicated that the minimum required sample size was three rats per group (https://powerandsamplesize.com/calculator/mean-2sample-2sided/).

Female Sprague-Dawley rats (21 days old) were purchased from Beijing HFK Biotechnology Co., Ltd. (Beijing, China). After 1 week of acclimatization, we established a rat model of PCOS using TP and a high-fat, high-sugar diet with some modifications to a previously reported method (Bisht et al., 2023; Ding et al., 2019). The high-fat, high-sugar diet used in this study (50.0% w/w standard feed, 16.8% lard, 19.2% sucrose, 9% casein, 1% minerals, 1% vitamins, and 3% maltodextrin) was based on a previously reported high-fat diet (Ding et al., 2019), replacing 4.2% of the standard feed with 4.2% sugar, as hyperglycemia can induce oxidative stress in the HA state (Gonzalez et al., 2012a; 2012b). TP and NAC were dissolved in propanediol and PBS, respectively. Because of differences in the reported intervention doses of NAC (100–1,000 mg/kg) in rats (Hu et al., 2021; Harada et al., 2003), we conducted the first batch of animal experiments to select the most appropriate NAC intervention dose. The rats were randomly allocated to eight groups (n = 5–7 in each group), with the following treatment conditions: Group 1 (control), fed standard chow and given the corresponding solvent treatment; Groups 2–4 (NAC intervention controls), fed standard chow and given daily oral gavage of 1 mL NAC (100, 200, or 400 mg/kg) with the corresponding propanediol treatment; Group 5 (PCOS model), fed high-fat, high-sugar diet, with daily subcutaneous injection of 0.25 mL TP (1 mg/kg) and the corresponding PBS treatment; Groups 6–8 (NAC intervention in PCOS model), fed high-fat, high-sugar diet, with daily subcutaneous injection of 0.25 mL TP (1 mg/kg) and oral gavage with 1 mL NAC (100, 200, or 400 mg/kg). Based on a preliminary analysis of rat-related data, we found that the 200 mg/kg NAC dose had the most favorable effect on metabolism and hormones. Therefore, we selected this dose as the main NAC intervention dose in rats and conducted a second batch of animal experiments. The rats were randomly allocated to four groups (n = 7 in each group) and treated as described above: Control, NAC intervention (200 mg/kg), PCOS, and NAC intervention (200 mg/kg) in the PCOS model. All groups were treated for 8 consecutive weeks, with weekly measurements of body weight. Dosages for oral gavage and subcutaneous injections were adjusted weekly based on recorded body weight changes. A schematic diagram of animal treatment process was shown in Figure 1A.

**FIGURE 1:**
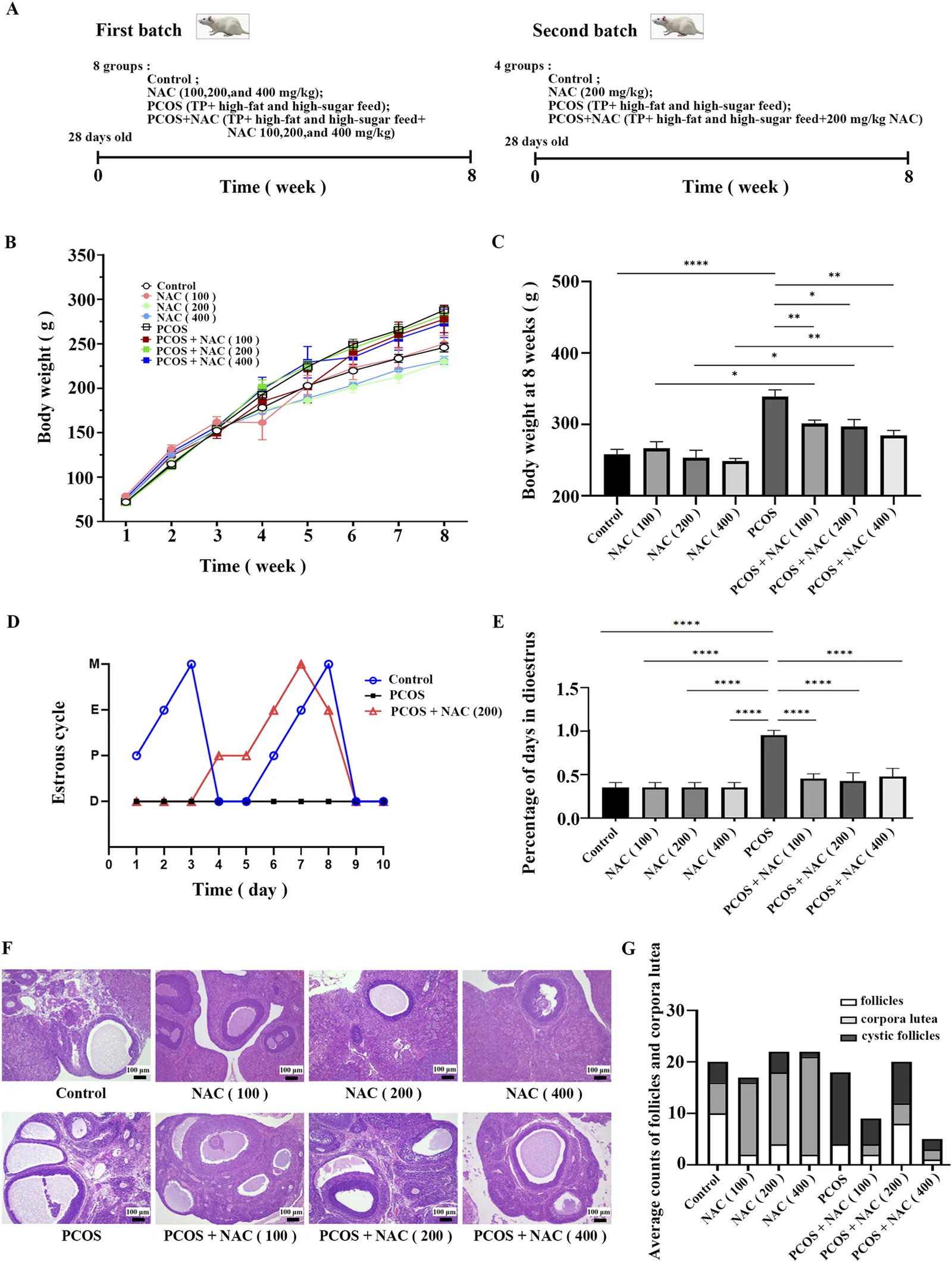
Effects of different doses (100, 200, and 400 mg/kg body weight) of N-acetylcysteine (NAC) on body weight, estrous cycle, and morphological changes in ovarian tissues in control and PCOS rats. **(A)** Schematic diagram of animal treatment process; **(B)** trend of body weight in rats during the intervention period and **(C)** body weight at 8 weeks of treatment (n = 4–14 rats in each group); **(D)** changes in the estrous cycle; **(E)** the percentage of days in dioestrus among groups (n = 4 rats in each group); **(F)** ovarian histopathology by H&E staining (100×, scale bar = 100 µm); and **(G)** proportion comparison of counts of developing follicles, corpora lutea, and cystic follicles in ovarian tissue among the different intervention rats (n = 4 rats in each group; Control vs. PCOS, χ^2^ = 14.061, *P* = 0.000; PCOS vs. PCOS + NAC200, χ^2^ = 6.884, *P* = 0.036). Data are represented as the mean ± standard deviation or proportion of counts. Significantly different, *P < 0.05, **P < 0.01, ***P < 0.001, ****P < 0.0001.

During the final 10 days of administration, the estrous cycles were monitored daily in four randomly selected rats per group based on vaginal smear staining results.

After 8 weeks of treatment, the rats were deeply anesthetized and euthanized after a 12-h fast.

Blood samples were collected from the abdominal aorta and centrifuged at 4 °C. Serum and plasma were aliquoted and stored at −80 °C for subsequent analysis.

Ovarian and hepatic tissues were collected and stored in PBS, liquid nitrogen, RNA stabilization solution, and 4% paraformaldehyde for flow cytometric detection of reactive oxygen species (ROS), Western blotting, RNA extraction, and histomorphological evaluation, respectively.

### 2.5 Analysis of serum hormones, hepatic enzymes, and metabolic parameters

Serum concentrations of luteinizing hormone (LH), follicle-stimulating hormone (FSH), T, and insulin (Ins) were measured using radioimmunoassays. SHBG, anti-Müllerian hormone (AMH), apolipoprotein (apo) A, apoB, and ox-LDL levels were determined using an ELISA (Shanghai XinFan Biotechnology, Shanghai, China). Alanine aminotransferase (ALT), aspartate aminotransferase (AST), and lactate dehydrogenase (LDH) were measured using kinetic methods (Zhejiang WeiYi Biotechnology, Zhejiang, China). Serum total cholesterol (TC), triglyceride (TG), LDL-C, HDL-C, and glucose (Glu) levels were measured using enzymatic methods.

The homeostatic model assessment of insulin resistance (HOMA-IR) was calculated using the following formula (Wang et al., 2025):

HOMA-IR = fasting Ins (μIU/mL) × fasting Glu (mmol/L)/22.5.

Insulin concentrations in SI units (pmol/L) were converted to conventional units (μIU/mL) by dividing by 6.945.

### 2.6 Quantification of ROS levels in rat hepatocytes

Hepatocytes were isolated from freshly excised rat liver tissue. Cellular ROS levels were measured by flow cytometry using 2′,7′-dichlorodihydrofluorescein diacetate as a fluorescent probe. Fluorescence intensity (488 nm excitation/530 nm emission) was quantified using a flow cytometer (Beckman CytoFLEX), with unstained cells used as negative controls. Data were analyzed using CytExpert software.

### 2.7 Histological sectioning and staining of rat liver and ovarian tissues

Liver and ovarian tissues from each rat group were fixed in 4% formaldehyde overnight at room temperature. According to the standard operating procedure for pathological examination, the tissues were dehydrated, trimmed, paraffin embedded, sectioned, and stained with hematoxylin (G1004, Servicebio, Wuhan, China) and eosin (YE2080, Bomei, Hefei, China) (H&E).

Liver tissues stored at −80 °C were trimmed, embedded in optimal cutting temperature compound, sectioned, and stained with Oil Red O (YO7512, Bomei, Hefei, China) and hematoxylin to reveal lipid droplets.

Images of the sections were collected and analyzed using a BA210Digital Trinocular Camera Microscopic Camera System (McDotti Industrial Group Co., Ltd.). The number of follicles and corpora lutea in the ovaries, as well as the extent of pathological changes in the liver, were evaluated using three randomly selected fields for each slice. Histopathological grading of steatosis, inflammation, and necrosis in the liver was performed according to the semiquantitative criteria defined in the International Harmonization of Nomenclature and Diagnostic Criteria for Rats and Mice (Mann, 2019). Each parameter above was graded on a five-tier scale: normal (−), minimal (+), mild (++), moderate (+++), or severe (++++). The lipid droplet area and percentage in the liver were analyzed using an image analysis system (Image-Pro Plus 6.0; Media Cybernetics, USA).

### 2.8 RNA extraction and analysis

Total RNA was extracted from the HepG2 cells and rat liver tissue using an RNA isolation NucleoSpin® RNA Plus kit (Macherey-Nagel, Germany). Reverse transcription (1.5 µg of total RNA in a total reaction volume of 15 µL) was performed using the PrimeScript Fast RT Reagent Kit with gDNA Eraser (TaKaRa Bio, Shiga, Japan). Quantitative real-time PCR amplification was performed with 28.6 ng of complementary DNA (cDNA) in a total reaction volume of 20 µL using TB Green™ Premix Ex1 Taq™ II (TaKaRa) with gene-specific primers (Supplementary Table S1) on a CFX Opus 96 Real-Time PCR System (Bio-Rad).

All reactions were performed in triplicate. Relative messenger RNA (mRNA) expression levels were calculated using the 2^−ΔΔCT^ method with normalization to 18S ribosomal RNA (rRNA).

### 2.9 Western blot analysis

Proteins were extracted from HepG2 cells and liver tissues using radioimmunoprecipitation assay (RIPA) buffer (Thermo Scientific, Rockford, IL, USA) with a complete phosphatase and protease inhibitor cocktail (MedChemExpress). Protein concentrations were determined using a bicinchoninic acid (BCA) protein assay kit. Subsequently, the proteins (the amount of protein loaded per lane: 50 µg of total protein for HepG2 cells and 35 µg of total protein for rat liver tissue) were separated by 12% sodium dodecyl sulfate-polyacrylamide gel electrophoresis (SDS-PAGE), transferred to polyvinylidene fluoride membranes (Millipore, USA), blocked with 5% milk, and incubated with the primary antibodies (Supplementary Table S2). After washing the membranes with Tris-buffered saline with Tween 20 (TBS-T), they were incubated with a horseradish peroxidase-conjugated secondary antibody (7074, 1:2000, Cell Signaling Technology) at room temperature for 1 h. Signals were detected using the Thermo Scientific™ Pierce™ enhanced chemiluminescence (ECL) Western blotting Substrate, and images were acquired with a ChemiDoc Imaging System (Bio-Rad). Signal intensities were quantified using ImageJ software (version 1.53a, NIH). Target protein expression levels were normalized to the corresponding loading PPIA controls.

### 2.10 Statistical analysis

Statistical analyses were performed using SPSS version 21.0 (IBM SPSS Statistics, Armonk, NY, USA). Differences among groups were assessed using one-way analysis of variance. For variables with homogeneous variances, differences between groups were compared using the least significant difference (LSD) method. Otherwise, the differences were evaluated using Dunnett’s T3 method. Chi-squared (χ^2^) test or Fisher’s exact probability was used to evaluate differences in percentages among the groups. Fisher’s exact test was used if there are more than 1 cell with a count less than 5 for χ^2^ test. Analysis of covariance was used to assess differences in variances after adjust for confounding factors such as animal experimental batch.

Longitudinal changes in rat body weight were analyzed using a linear mixed-effects model, with time, group, and time × group interaction as fixed effects and individual rat as a random effect, in GraphPad Prism (version 8.0.2.263). Post hoc pairwise comparisons between groups at individual time points were performed using Sidak’s multiple comparisons test.

The data is represented as mean ± standard deviation unless otherwise stated. *P* < 0.05 was considered statistically significant.

## 3 Results

### 3.1 TP suppresses SHBG expression and secretion through HNF4α downregulation in HepG2 cells, similarly to oxidized lipoproteins

TP treatment significantly suppressed both mRNA expression and protein levels of SHBG and HNF4α relative to controls (Figures 2A,B,E–G; *P* < 0.05). Lipoprotein and oxidized lipoprotein treatments differentially downregulated SHBG/HNF4α mRNA and protein, with ox-HDL and ox-LDL exhibiting stronger inhibition (Figures 2A,B,E–G; *P* < 0.05). Ox-LDL increased the expression of *PPAR-γ* mRNA (Figure 2D; *P* < 0.05), but not protein (Figure 2I; *P* > 0.05). No significant effects of TP, lipoproteins, or oxidized lipoproteins were observed on the mRNA or protein levels of COUP-TF1 (Figures 2C,F,H; *P* > 0.05).

**FIGURE 2:**
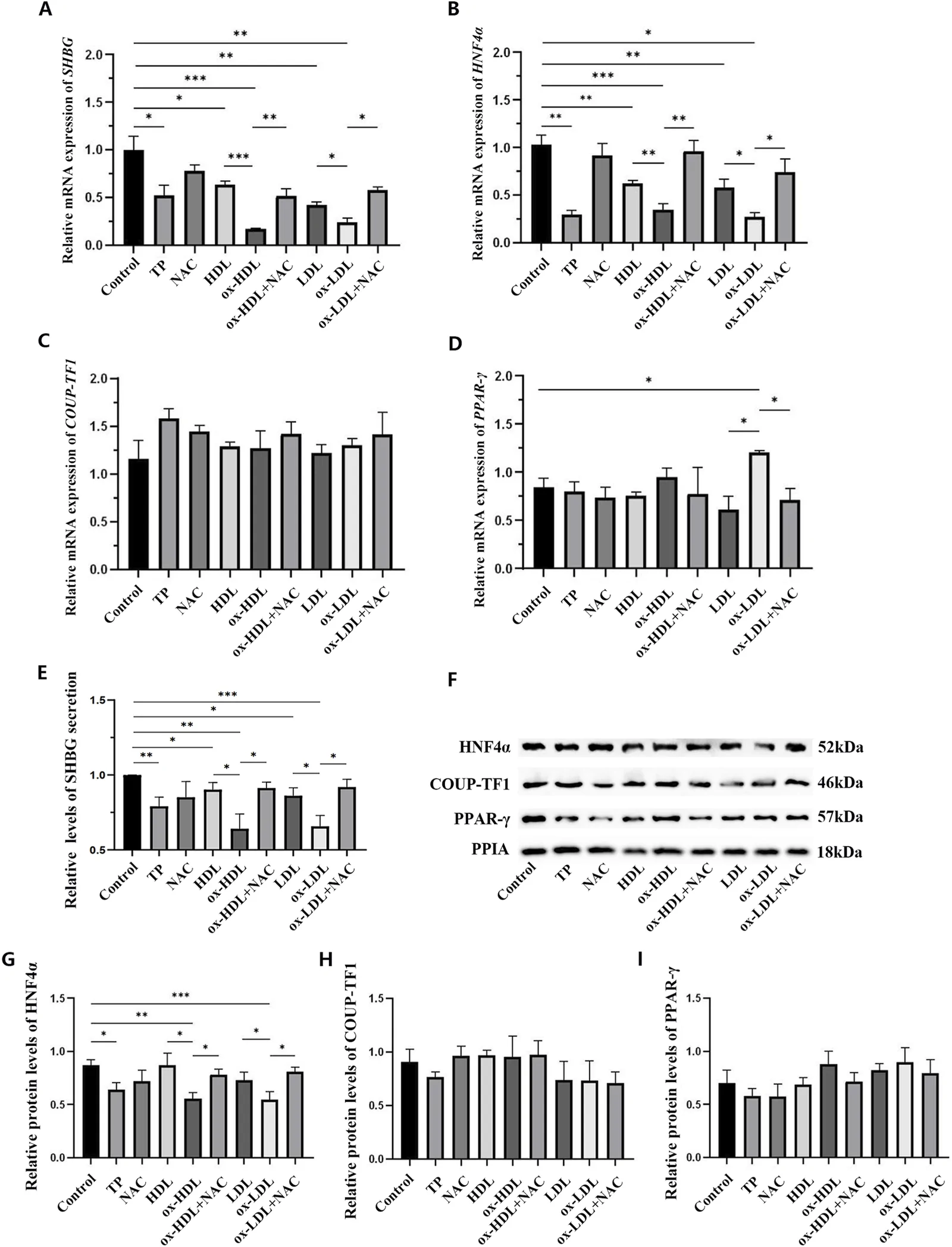
Effects of testosterone propionate (TP), oxidized lipoproteins, and oxidized lipoproteins plus N-acetylcysteine (NAC) treatment on the expression of sex hormone-binding globulin (SHBG) and its main transcriptional regulatory factors at 48 h in HepG2 cells. The relative mRNA expression for **(A)** *SHBG*, **(B)** hepatocyte nuclear factor-4α (*HNF4α*), **(C)** chicken ovalbumin upstream promoter-transcription factor 1 (*COUP-TF1*), and **(D)** peroxisome proliferator-activated receptor-γ (*PPAR-γ*), with *18S* used as an internal control (n = 3 in each group). **(E)** Relative SHBG secretion in the culture supernatant (normalized to total cellular protein, n = 6 in each group). **(F)** Representative western blots and relative protein levels of **(G)** HNF4α, **(H)** COUP-TF1, and **(I)** PPAR-γ in HepG2 cells, with peptidylprolyl isomerase A (PPIA) used as an internal control (n = 4 in each group). HDL, high-density lipoprotein; ox-HDL, oxidized HDL; LDL, low-density lipoprotein; ox-LDL, oxidized LDL. Data are represented as the mean ± standard deviation from three to six independent experiments. Significantly different, *P < 0.05, **P < 0.01, ***P < 0.001.

### 3.2 NAC ameliorates oxidized lipoprotein-induced suppression of SHBG/HNF4α in HepG2 cells

NAC treatment significantly increased *SHBG* and *HNF4α* mRNA expression and elevated SHBG secretion and HNF4α protein levels under ox-HDL or ox-LDL treatment conditions in HepG2 cells (Figures 2A,B,E–G; *P* < 0.05). In contrast, NAC had no significant effect on COUP-TF1 mRNA, or COUP-TF1 and PPAR-γ protein levels in NAC treated control cells and oxidized lipoprotein-treated cells (Figures 2C,F,H, and I; *P* > 0.05); however, NAC reversed the ox-LDL-induced increase in PPAR-γ mRNA expression (Figure 2D; *P* < 0.05).

### 3.3 Successful establishment of a PCOS rat model with TP and a high-fat and high-sugar diet

As shown in Figure 1B, rat body weight significantly changed over time (time effect: *P* < 0.0001) and differed among experimental groups (group effect: *P* < 0.0002). A significant time-by-group interaction was observed (*P* < 0.0001), indicating that the trajectories of body weight change differed among groups. PCOS rats exhibited a significantly higher body mass than controls from week 6 onward throughout the study period (*P* < 0.05). At week 8, the PCOS group had the highest body weight among all groups (Figure 1C). Estrous cycle analysis revealed persistent diestrus in PCOS rats (Figures 1D,E). Histopathological evaluation showed abundant cystic follicles with reduction in developing follicles and corpora lutea in the ovaries of PCOS rats than in that of the control rats (Figures 1F,G; *P* < 0.001). H&E-stained hepatic sections from untreated PCOS rats demonstrated minimal inflammatory cell infiltration, minimal and sporadic punctate cell necrosis, and extensive microvesicular steatosis (Figures 3A,C). Oil Red O staining showed that the PCOS group had the highest percentage of lipid droplet area in liver tissue among all groups (Figures 3B,D). PCOS rats also showed increased serum T concentrations, LH/FSH ratio, fasting Ins and Glu, TG, ox-LDL, and HOMA-IR (*P* < 0.05), but decreased FSH, SHBG, HDL-C, and apoA levels compared with the untreated control rats (Table 2; *P* < 0.05).

**FIGURE 3:**
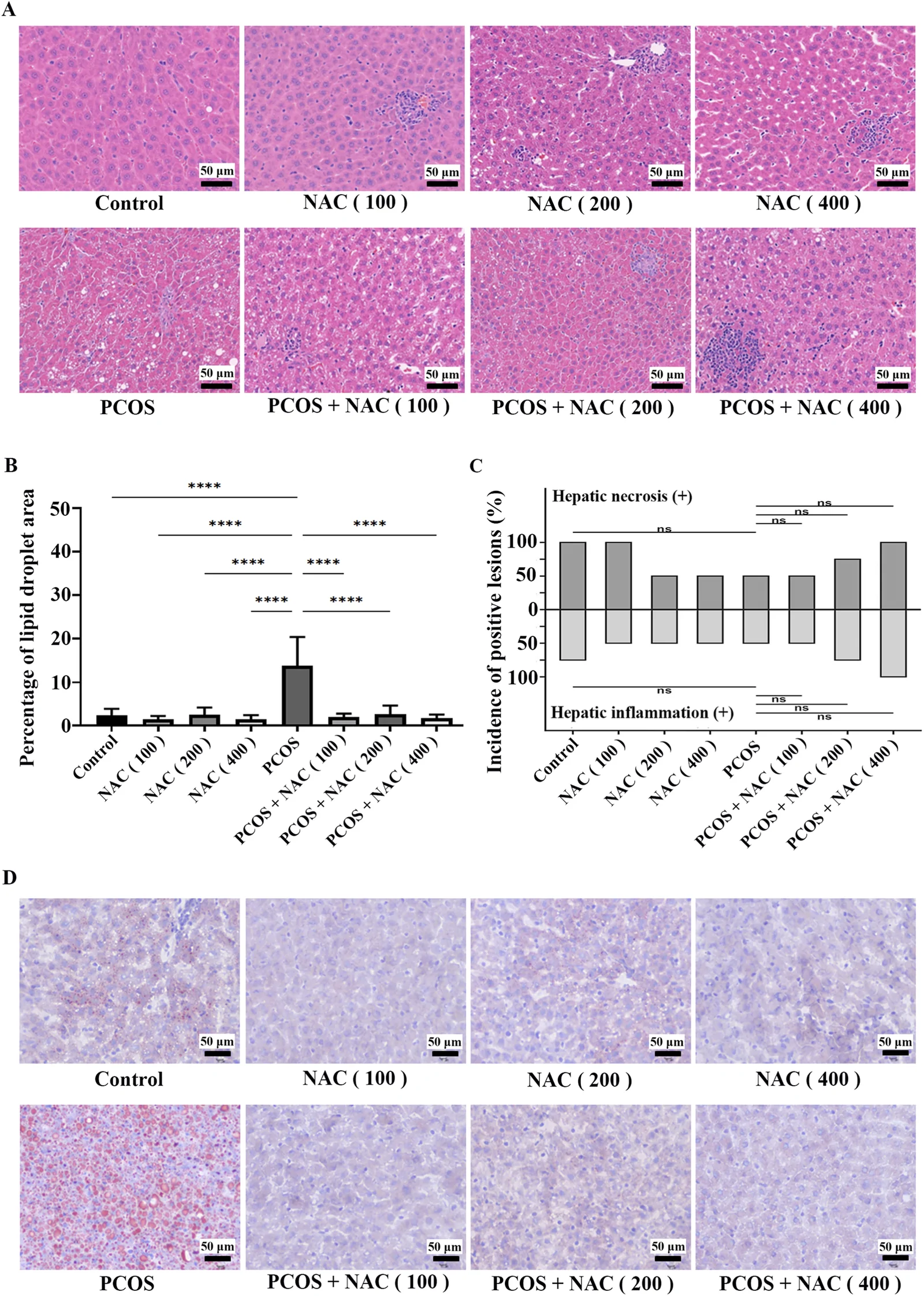
Effects of different doses (100, 200, and 400 mg/kg body weight) of N-acetylcysteine (NAC) on liver histomorphology in control and PCOS rats. **(A)** Liver histopathology by H&E staining (400×, scale bar = 50 µm); **(B)** percentage of lipid droplet area in liver tissue (n = 4 rats in each group); **(C)** incidence of positive necrosis or inflammatory lesions in liver tissue (n = 4 rats in each group, all observed positive lesions were graded as minimal); and **(D)** Oil red O staining of liver tissue (400×, scale bar = 50 µm). Data are represented as the mean ± standard deviation unless otherwise stated. ns, not significant. Significantly different, ****P < 0.0001.

**TABLE 2:** Levels of serum hormones, glucose, lipids, and liver enzymes in control and PCOS rats.

Parameters	Control	NAC (100)	NAC (200)	NAC (400)	PCOS	PCOS NAC (100)	PCOS NAC (200)	PCOS NAC (400)
n	13	5	13	5	14	7	14	4
AMH (µg/L)	2.08 ± 0.39	2.11 ± 0.33	2.04 ± 0.40	2.13 ± 0.19	2.28 ± 0.48	2.12 ± 0.38	1.96 ± 0.44^b^	1.96 ± 0.18
LH (IU/L)	2.84 ± 0.62	2.23 ± 0.82	2.70 ± 1.11	2.35 ± 0.53	3.02 ± 0.96	3.07 ± 0.27	2.96 ± 0.53	2.87 ± 1.06
FSH (IU/L)	2.22 ± 0.36	1.56 ± 0.46	2.06 ± 0.55	1.38 ± 0.12^a^	1.78 ± 0.35^a^	1.51 ± 0.28^a^	2.28 ± 0.73^b^	1.69 ± 0.16
LH/FSH	1.26 ± 0.41	1.57 ± 0.08	1.23 ± 0.38	1.68 ± 0.33	1.83 ± 0.67^a^	2.08 ± 0.33^a^	1.33 ± 0.37^b^	1.71 ± 0.59
T (nmol/L)	0.14 ± 0.06	0.20 ± 0.02	0.20 ± 0.09	0.20 ± 0.05	2.70 ± 1.73^a^	1.52 ± 0.88^a,b^	1.51 ± 1.61^a,b^	1.72 ± 0.58^a^
SHBG (nmol/L)	76.34 ± 12.52	76.50 ± 14.68	76.67 ± 9.41	75.08 ± 9.50	66.92 ± 9.04^a^	79.83 ± 14.21	77.85 ± 8.57^b^	80.04 ± 9.31
Fasting Glu (mmol/L)	8.14 ± 0.68	11.22 ± 1.24^a^	8.26 ± 0.65	10.48 ± 0.69^a^	9.31 ± 0.94^a^	10.63 ± 0.87^a,b^	9.50 ± 0.79^a^	12.30 ± 0.83^a,b^
Fasting Ins (pmol/L)	119.28 ± 21.24	175.29 ± 55.25^a^	112.87 ± 24.18	178.54 ± 19.21^a^	185.31 ± 92.54^a^	174.42 ± 52.48^a^	152.38 ± 42.96 0.55^a^	185.83 ± 47.95^a^
HOMA-IR	6.40 ± 1.43	12.41 ± 3.42^a^	6.01 ± 1.59	11.94 ± 1.12^a^	11.29 ± 6.96^a^	12.01 ± 4.15^a^	9.35 ± 3.06^a^	14.56 ± 3.56^a^
TG (mmol/L)	0.44 ± 0.21	0.73 ± 0.37^a^	0.54 ± 0.18	0.58 ± 0.19	1.02 ± 0.17^a^	0.91 ± 0.46^a^	1.00 ± 0.27^a^	1.17 ± 0.49^a,b^
TC (mmol/L)	1.42 ± 0.21	1.39 ± 0.14	1.48 ± 0.21	1.18 ± 0.30	1.35 ± 0.15	1.47 ± 0.19	1.53 ± 0.21^b^	1.07 ± 0.04^a,b^
HDL-C (mmol/L)	0.43 ± 0.07	0.41 ± 0.04	0.39 ± 0.04	0.36 ± 0.11	0.33 ± 0.04^a^	0.36 ± 0.09	0.39 ± 0.05^b^	0.26 ± 0.02^a,b^
LDL-C (mmol/L)	0.10 ± 0.04	0.09 ± 0.04	0.09 ± 0.03	0.08 ± 0.03	0.12 ± 0.03	0.17 ± 0.03^a,b^	0.14 ± 0.03^a^	0.15 ± 0.04^a^
ApoA (mg/L)	4.66 ± 0.90	4.65 ± 0.78	4.81 ± 0.79	4.22 ± 0.53^a^	3.80 ± 0.78^a^	4.23 ± 0.67^a^	4.99 ± 0.74^b^	5.01 ± 0.40
ApoB (mg/L)	200.43 ± 41.32	179.26 ± 28.34^a^	190.30 ± 28.78	163.34 ± 22.43^a^	183.71 ± 40.71	190.49 ± 29.40	187.54 ± 27.78	193.62 ± 22.06
Ox-LDL (mg/L)	4.57 ± 0.89	4.45 ± 0.78	4.59 ± 0.86	4.77 ± 0.52	5.47 ± 0.76^a^	4.84 ± 0.87	4.84 ± 0.50	4.77 ± 0.55
ALT (U/L)	40.00 ± 8.12	39.20 ± 8.04	44.23 ± 9.55	49.60 ± 19.87	44.50 ± 6.27	42.29 ± 10.08	48.53 ± 11.56^a^	44.50 ± 4.80
AST (U/L)	78.57 ± 10.32	70.00 ± 8.94	81.92 ± 11.67	81.40 ± 17.57	72.00 ± 9.07	65.71 ± 8.64^a^	72.60 ± 9.33	74.00 ± 13.37
LDH (U/L)	421.55 ± 41.09	218.40 ± 59.16^a^	408.30 ± 179.72	308.60 ± 85.40	407.27 ± 174.61	207.14 ± 61.80^a,b^	414.60 ± 171.79	276.25 ± 55.95

Values are presented as the mean ± standard deviation.

AMH, anti-Müllerian hormone; ApoA, apolipoprotein A; ApoB, apolipoprotein B; ALT, alanine aminotransferase; AST, aspartate aminotransferase; FSH, follicle-stimulating hormone; Glu, glucose; HDL-C, high-density lipoprotein; HOMA-IR, the homeostatic model assessment of insulin resistance; Ins, insulin; LDH, lactate dehydrogenase; LDL-C, low-density lipoprotein; LH, luteinizing hormone; Ox-LDL, oxidized LDL; SHBG, sex hormone-binding globulin; TC, total cholesterol; TG, triglycerides; T, testosterone.

Comparisons of all parameters between the two groups were corrected for differences in experimental batch.

^a^ *P* < 0.05, compared to the control group.

^b^ *P* < 0.05, compared to the PCOS group.

### 3.4 NAC partially ameliorates obesity and hormonal and metabolic abnormalities, restores estrous cyclicity and ovarian morphology, and improves hepatic steatosis in PCOS rats

As illustrated in Figure 1C, NAC treatment significantly reduced the body weight of rats with PCOS (*P* < 0.05). Estrous cycle analysis revealed a partial restoration of cyclicity in NAC-treated PCOS rats, although the cycle length remained prolonged (6–7 days) (Figures 1D,E). Ovarian histology revealed an increased the number of developing follicles and corpora lutea, along with reduced cystic follicle formation, in rats treated with 200 mg/kg NAC compared with the untreated PCOS rats (Figures 1F,G; *P* < 0.05). The initial histopathological semi-quantitative grading assessment of rat liver tissue revealed mild steatosis, minimal inflammatory cell infiltration, and minimal and sporadic punctate necrosis across all groups based on H&E-stained sections (Figure 3A). No statistical differences were observed in the incidences of positive necrosis and inflammatory lesions among all groups (Figure 3C; *P* > 0.05). Further lipid quantification based on Oil Red O-stained sections showed that the percentage of lipid droplet area was significantly decreased in the three NAC-treated PCOS groups compared with that in the untreated PCOS group (*P* < 0.001), and was similar between the three NAC-treated non-PCOS groups and the untreated controls (*P* > 0.05) (Figures 3B,D).

As described in Table 2, compared with the PCOS rats, 100 mg/kg NAC significantly decreased the serum T and LDH levels (*P* < 0.05), and increased the fasting Glu and LDL-C levels (*P* < 0.05); 200 mg/kg NAC decreased the circulating AMH and T concentrations and LH/FSH ratio (*P* < 0.05), and increased the FSH, SHBG, TC, HDL-C, and apoA levels (*P* < 0.05); 400 mg/kg NAC increased the serum fasting Glu and TG levels (*P* < 0.05), and reduced the TC and HDL-C levels (*P* < 0.05) in rats with PCOS. Notably, compared with control rats, NAC treatment increased fasting Glu, fasting Ins, TG, and HOMA-IR, and decreased FSH, LDH, apoA, and apoB levels at 100 and/or 400 mg/kg in the non-PCOS groups (Table 2; *P* < 0.05). Overall, 200 mg/kg NAC produced the most favorable effects on the hormone profile and glycolipid metabolism among the three doses tested in rats with PCOS.

### 3.5 NAC attenuates hepatic oxidative stress by reducing ROS and may upregulate SHBG production by affecting HNF4α/COUP-TF1/PPAR-γ expression in PCOS rats

PCOS rats exhibited significantly elevated liver ROS levels compared with control rats (Figures 4A,E,I; *P* = 0.0002). Compared with the untreated PCOS rats, NAC significantly reduced ROS levels at 200 mg/kg (Figures 4E,G,I; *P* = 0.0013), but not at 100 or 400 mg/kg (Figures 4E,F,H,I; *P* > 0.05).

**FIGURE 4:**
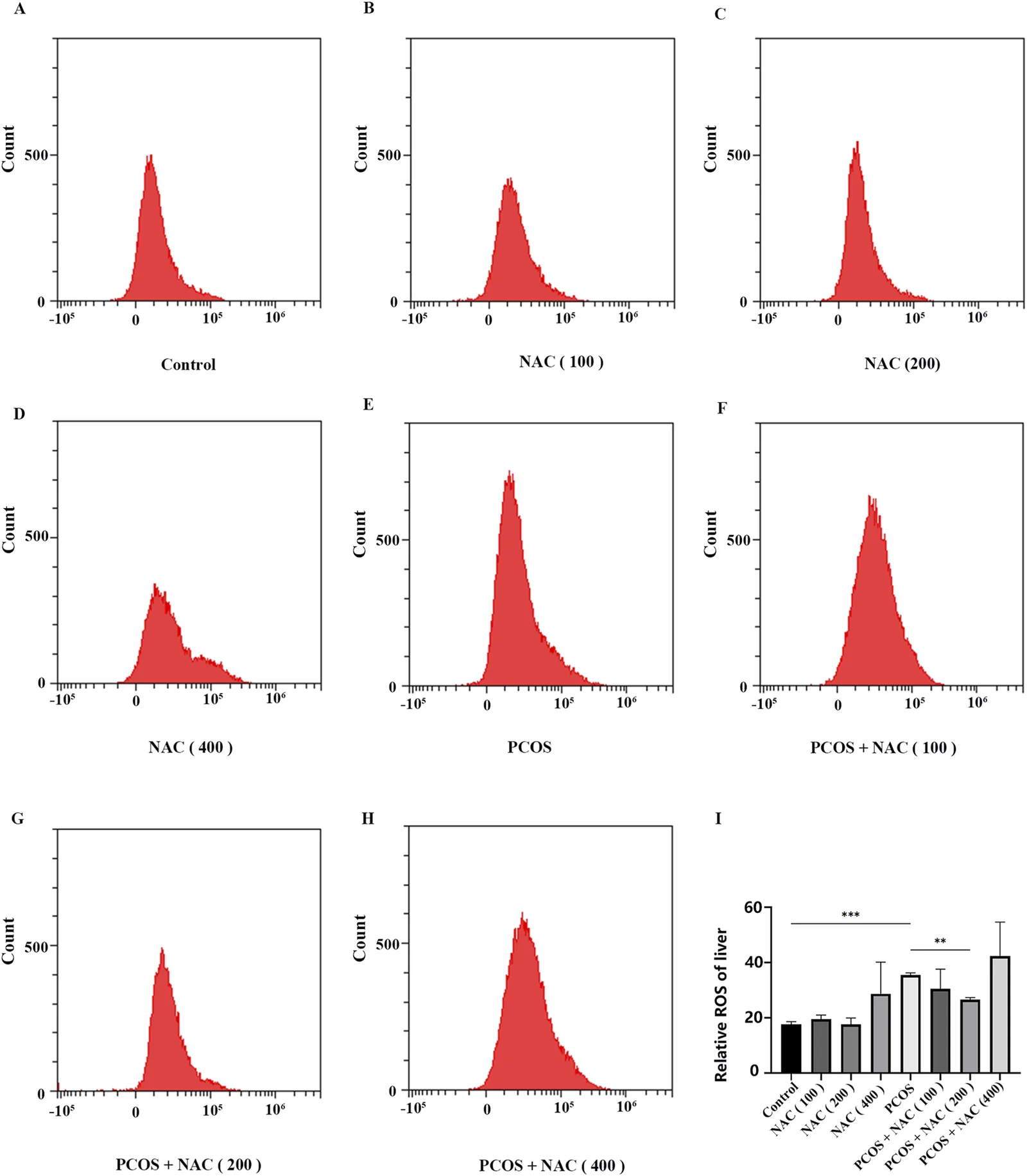
Effects of different doses (100, 200, and 400 mg/kg body weight) of N-acetylcysteine (NAC) on hepatic reactive oxygen species (ROS) levels in control and PCOS rats. The ROS levels in hepatocytes of rats: **(A)** control, **(B)** NAC (100), **(C)** NAC (200), **(D)** NAC (400), **(E)** PCOS, **(F)** PCOS + NAC (100), **(G)** PCOS + NAC (200), **(H)** PCOS + NAC (400). **(I)** Comparison of the hepatic ROS levels (fluorescence intensities) among the different intervention groups (n = 4 rats in each group). Data are represented as the mean ± standard deviation. Significantly different, **P = 0.0013, ***P = 0.0002.

At the molecular level, PCOS rats had significantly lower mRNA expression of *Shbg*, and *Hnf4α*, and lower protein levels of SHBG and HNF4α (Figures 5A,B,E,F; *P* < 0.01), accompanied by elevated *Ppar-γ* mRNA expression and COUP-TF1 protein levels (Figures 5D,G; *P* < 0.05) in the liver. NAC treatment partially or completely reversed these alterations, increasing SHBG/HNF4α expression (Figures 5A,B,E,F; *P* < 0.05), but decreasing COUP-TF1/PPAR-γ expression (Figures 5C,D,G,H; *P* < 0.05) at the mRNA and protein levels. Notably, NAC treatment at 400 mg/kg induced paradoxical effects characterized by increased *Shbg* mRNA (*P* = 0.0028) and decreased SHBG protein (*P* = 0.0467) levels in PCOS rats. Additionally, compared with the control rats, NAC treatment at 100, 200, and/or 400 mg/kg also decreased the mRNA expression of *Shbg*, *Hnf4α*, *Coup-tf1*, and Ppar-γ (Figures 5A–D) and the protein levels of COUP-TF1 (Figure 5G) in the non-PCOS rats (*P* < 0.05).

**FIGURE 5:**
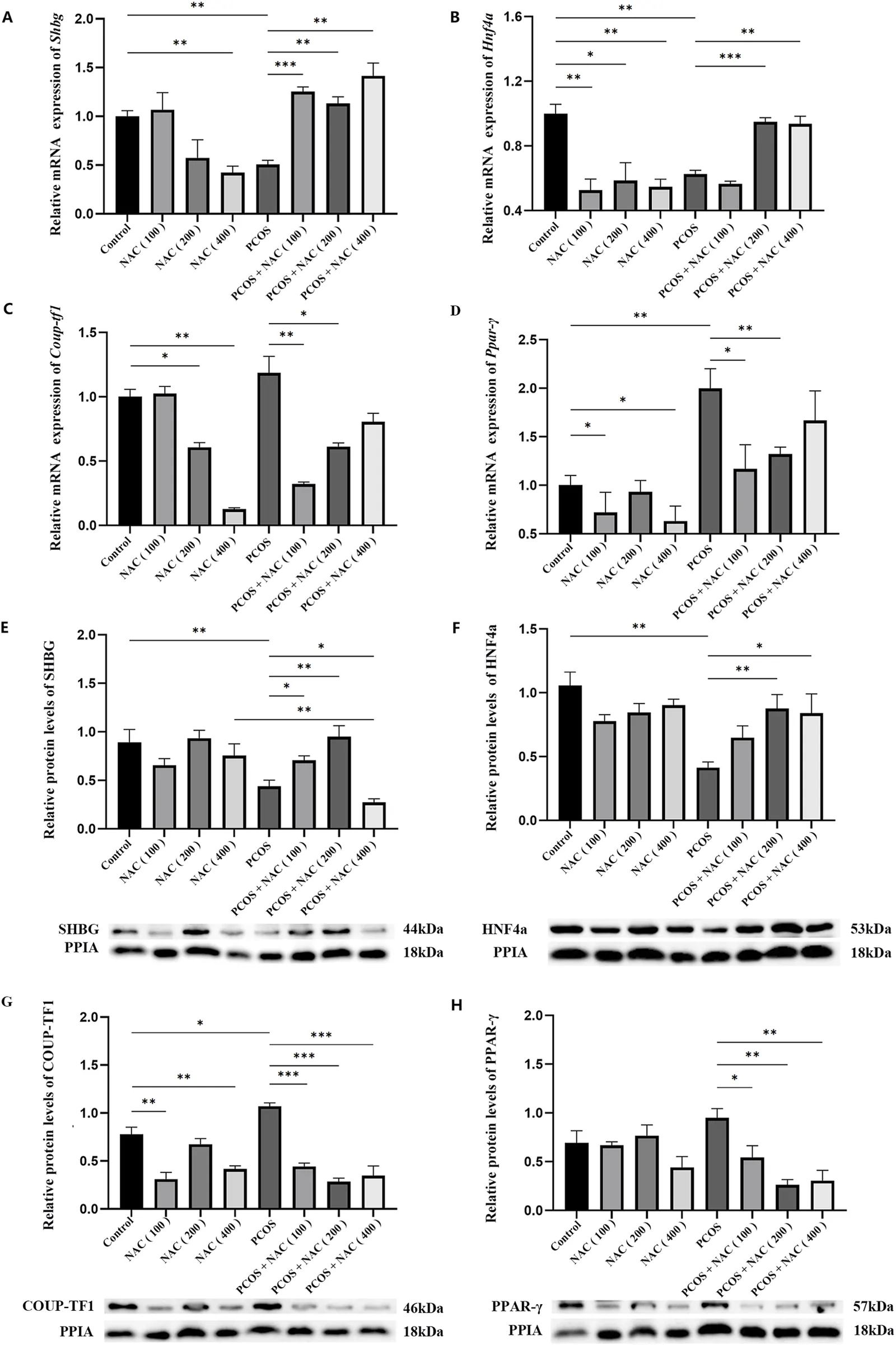
Effects of different doses (100, 200, and 400 mg/kg body weight) of N-acetylcysteine (NAC) on the expression of sex hormone-binding globulin (SHBG) and its main transcription regulatory factors in the rat liver. The relative mRNA expression for **(A)** *Shbg*, **(B)** hepatocyte nuclear factor 4α (*Hnf4α*), **(C)** chicken ovalbumin upstream promoter-transcription factor 1 (*Coup-tf1*), and **(D)** peroxisome proliferator-activated receptor-γ (*Ppar-γ*), with *18S* used as an internal control. The results of Western blotting and the relative protein levels for **(E)** SHBG, **(F)** HNF4α, **(G)** COUP-TF1, and **(H)** PPAR-γ, with peptidylprolyl isomerase A (PPIA) used as an internal control. Data are represented as the mean ± standard deviation (n = 4 rats in each group). Significantly different, *P < 0.05, **P < 0.01, ***P < 0.001.

In summary, among the three doses tested in PCOS rats, NAC showed the most favorable effect at 200 mg/kg in reducing hepatocyte ROS levels and increasing SHBG expression.

## 4 Discussion

We previously revealed that oxidative stress (ox-LDL and ox-HDL) inhibits the production and secretion of SHBG by downregulating the expression of HNF4α in HepG2 cells, which may be a significant factor in aggravating hyperandrogenemia in PCOS (Sun et al., 2021). In this study, we found that NAC treatment significantly ameliorated downregulation of SHBG and HNF4α induced by ox-LDL and ox-HDL in HepG2 cells. We also found that androgen (TP) may suppress SHBG secretion by downregulating HNF4α expression in HepG2 cells. Furthermore, we found that NAC intervention ameliorated obesity, hyperandrogenemia, and dyslipidemia, reduced hepatocyte ROS levels, improved estrous cyclicity and follicular development, and may restore hepatic SHBG production by upregulating HNF4α and downregulating COUP-TF1/PPAR-γ expression in PCOS rat models, with the 200 mg/kg showing the most favorable overall effect among the three doses tested.

HA is the core pathological feature of PCOS (Teede et al., 2023; Escobar-Morreale, 2018; Rosenfield and Ehrmann, 2016). SHBG plays a crucial role in regulating the stability of sex hormones and the levels of bioactive androgens by binding to and transporting these hormones in circulation (Szybiak-Skora et al., 2025; Aydin and Winters, 2025; Zhu et al., 2019). Low SHBG levels aggravate hyperandrogenemia and are associated with an increased risk of several metabolic disorders (Szybiak-Skora et al., 2025; Zhu et al., 2019). In contrast, hyperandrogenemia can trigger a self-amplifying cycle, wherein androgens suppress hepatic SHBG production, as evidenced clinically (Aydin and Winters, 2025; Zhu et al., 2019; Plymate et al., 1983), although testosterone has been reported to stimulate the production and secretion of SHBG under specific conditions (supraphysiological concentrations) in HepG2 cells (Loukovaara et al., 1995). In this study, we demonstrated that androgen (TP) treatment significantly reduced SHBG secretion, which may be associated with reduced HNF4α mRNA and protein levels, with no significant effect on the expression of *COUP-TF1* and *PPAR-γ*.

Oxidative stress is an imbalance between prooxidants and antioxidants in the body (Zhang et al., 2017; Murri et al., 2013). Patients with PCOS often exhibit increased oxidative stress (Zhang et al., 2024; Sun et al., 2021; Zhang et al., 2017; Murri et al., 2013; Gonzalez et al., 2012b). Studies have shown that genetic variants of oxidative stress-related genes, including the A allele of A16V single nucleotide polymorphism (SNP) in superoxide dismutase 2 gene, the T allele of G994T SNP in platelet-activating factor acetylhydrolase gene, the L allele of P198L SNP in glutathione peroxidase 1 gene, the RR genotype of Q192R variant in paraoxonase 1 gene, the T allele of T-413A SNP in heme oxygenase-1 gene, the A allele of G-463A SNP in myeloperoxidase gene, and the T allele of C-1054T variant in cytochrome P450 2E1 gene, are associated with an increased risk of developing PCOS (Wang et al., 2025; Pu et al., 2023; Ma et al., 2021; Sun et al., 2019; Liu et al., 2019; Wang et al., 2012; Fan et al., 2010), confirming that women with PCOS exhibit a genetic predisposition to oxidative stress. Therefore, the inflammatory state and hormonal and metabolic abnormalities, including inflammatory cytokines, HA, IR, hyperglycemia, and dyslipidemia, initiate and exacerbate oxidative stress (Liu et al., 2021; Gonzalez et al., 2020; Bannigida et al., 2020; Haghikia and Landmesser, 2018; Zhang et al., 2017; Zhang et al., 2015).

HA and oxidative stress are mutually reinforcing, forming a vicious cycle. Androgens can sensitize circulating leukocytes to glucose and accelerate ROS production in non-obese women with PCOS and lean healthy women of reproductive-age (Gonzalez et al., 2012b; Gonzalez et al., 2012a). Women with PCOS and HA exhibit more severe oxidative stress and impaired HDL antioxidant and anti-inflammatory functions than women without HA (Zhang et al., 2017; Zhang et al., 2015). Conversely, oxidative stress has been shown to inhibit the secretion of SHBG by downregulating HNF4α in HepG2 cells and to promote the production of testosterone by upregulating the expression of key androgen-synthesis enzymes and proteins in rat ovarian theca–interstitial cells (Zhang et al., 2024; Sun et al., 2021), suggesting that it may be a key factor involved in exacerbating hyperandrogenemia in PCOS.

The antioxidant NAC reduces androgen levels and ROS production, increases insulin sensitivity, and improves metabolic abnormalities, mitochondrial function, and reproductive outcomes in women with PCOS and in animal models of PCOS (Vina et al., 2025; Fang et al., 2024; Shahveghar Asl et al., 2023; Liu et al., 2023; Yao et al., 2022; Sandhu et al., 2021). However, relatively little research has examined the relationship between NAC and SHBG, and the results have been inconsistent. NAC treatment has been reported to increase (Oner and Muderris, 2011; Fulghesu et al., 2002), decrease (Nemati et al., 2017), or have no effect (Vina et al., 2025) on circulating SHBG levels in patients with PCOS. In this study, we found that NAC treatment attenuated the decrease in SHBG secretion induced by ox-LDL and ox-HDL by upregulating the expression of HNF4α in HepG2 cells. We further demonstrated that NAC intervention (200 mg/kg dose) significantly elevated hepatic SHBG mRNA and protein levels, possibly by upregulating HNF4α and downregulating COUP-TF1/PPAR-γ expression (mRNA and protein) in the PCOS rat model. We also showed that NAC intervention in PCOS rats significantly reduced body weight, circulating testosterone concentrations, LH/FSH ratio, hepatic ROS levels, and hepatic lipid deposition; elevated serum SHBG, TC, HDL-C, and apoA levels; and partially restored estrous cyclicity and ovarian follicular development. Our findings suggest that NAC not only has a beneficial effect on hormones, metabolism, oxidative stress, and ovarian function, but also increases hepatic SHBG production and secretion by affecting the expression of the transcription factors HNF4α, COUP-TF1, and PPAR-γ, thereby improving hyperandrogenemia in PCOS.

Reports of postnatal hepatic SHBG expression in rodents are inconsistent. Using transgenic mouse models, Reventós et al. (1993) demonstrated that the rat *Shbg* gene is transiently expressed in the fetal liver, but undergoes transcriptional silencing after birth, with circulating SHBG in adulthood derived primarily from the gonads. Jänne et al. (1999) further supported this hypothesis, suggesting that wild-type rodent models are generally unsuitable for studying hepatic SHBG regulation. However, Li et al. (2015) found age-dependent increases in SHBG expression in the liver, brain, and prostate of male Sprague-Dawley rats. Several recent studies have demonstrated that rat hepatic cells express SHBG in PCOS rat models (Xing et al., 2021; Zhao et al., 2023; Hu et al., 2024). Our findings, which support these reports, indicate that SHBG mRNA and protein expression can be detected in the liver of rats with or without PCOS.

The effects of oxidative stress and NAC treatment on SHBG expression may involve different mechanisms in HepG2 cells and the PCOS rat model. In this study, among the three transcription factors regulating SHBG expression, oxidative stress and NAC treatment did not affect the expression of COUP-TF1/PPAR-γ in HepG2 cells, but significantly affected the expression of these two transcription factors in rats with PCOS. A previous report showed that the oxidative stress-induced decrease in SHBG expression is mediated mainly by HNF4α rather than COUP-TF1 or PPAR-γ (Sun et al., 2021), supporting the cell findings in this study. COUP-TF1 can compete with HNF-4α for binding to the promoter region of the SHBG gene. When HNF4α levels decline in obesity, lipogenesis increases and fatty liver develops; COUP-TF1 then binds to this site and blocks SHBG transcription (Zhu et al., 2019). PPAR-γ has been reported to regulate adipocyte differentiation, adipogenesis, and lipid uptake and storage and to improve insulin sensitivity and alleviate inflammatory responses (Soares et al., 2024). The expression of SHBG and HNF4α was decreased and the protein levels of PPAR-γ were increased, whereas pioglitazone or metformin treatment elevated SHBG and HNF4α but not PPAR-γ expression in the liver of rats with PCOS and IR (Zhao et al., 2023). The difference between the animal and cell findings may reflect complex metabolic processes, particularly abnormalities in lipid metabolism and related regulation in the animal model. Further studies are required to clarify the differences between these mechanisms.

At 400 mg/kg, NAC intervention in PCOS rats increased hepatic *Shbg* mRNA and serum SHBG levels but decreased hepatic SHBG protein levels relative to untreated PCOS rats. This discordance may reflect translational or post-translational regulation, altered secretion, serum kinetics, biological variability, or differences among qPCR, Western blotting, and ELISA measurements of SHBG. Moreover, in addition to hepatic SHBG, circulating SHBG in adult rats may originate from other organs such as the gonads, brain, kidneys, and prostate (Li et al., 2015; Janne et al., 1999; Reventos et al., 1993). Additionally, due to the fact that the PCR amplification products of rat *Shbg* do not include exon 1 in this study, they may not be able to distinguish transcripts originating from different first exons. Further studies are needed to validate this phenomenon and explain the underlying mechanisms.

Maintenance of redox balance through various adaptive response mechanisms is crucial for normal metabolic regulation and biological functions (Sies et al., 2022). Appropriate oxidant levels are essential for sustaining physiological redox signal transduction (Sies et al., 2022; Sies, 2017). Excessive oxidant production and/or insufficient antioxidant capacity can impair the structure and function of biomolecules, leading to disrupted redox signaling (Sies et al., 2022). Conversely, insufficient oxidant levels can impair physiological oxidative signaling, resulting in cellular dysfunction and reductive stress (Manford et al., 2020; Handy and Loscalzo, 2017). In this study, we found that NAC treatment at doses of 100, 200, and 400 mg/kg did not further increase hepatic SHBG expression, but increased fasting Glu and Ins levels and induced IR in non-PCOS rats, supporting another recent study showing that NAC intervention in healthy male mice has adverse effects on offspring development and placental structure (Derrico et al., 2025). In PCOS rats, NAC treatment increased fasting Glu levels at doses of 100 or 400 mg/kg compared with those untreated PCOS rats. This dose-dependent divergence may be related to insufficient or excessive antioxidant activity. Overall, antioxidant therapy requires precise redox balance (Sies et al., 2022) because insufficient or excessive use of antioxidants may lead to suboptimal or adverse effects.

This study has four strengths. (i) This is the first study to report that NAC treatment can restore SHBG expression under oxidative stress conditions in HepG2 cells and in a rat model of PCOS induced by TP and a high-fat, high-sugar diet. (ii) The restoration of SHBG expression by NAC treatment may be associated with HNF4α upregulation in HepG2 cells and with both HNF4α upregulation and COUP-TF1/PPAR-γ downregulation in PCOS rat models. (iii) In the animal experiments, we included three NAC treatment doses and corresponding NAC-treated non-PCOS control groups. The results showed that 200 mg/kg NAC reduced hepatic ROS levels, increased SHBG expression, and improved hormonal profiles and lipid metabolism in rats with PCOS. However, NAC intervention at 100 and 400 mg/kg had less favorable effects and was associated with adverse effects on glucose metabolism in NAC-treated non-PCOS rats. These findings indicate the importance of precise redox regulation and suggest that NAC may have different effects under non-PCOS conditions. (iv) We also demonstrated that androgen (TP) may reduce SHBG production by downregulating the expression of HNF4α in HepG2 cells.

The present study has several limitations. First, the findings were derived from *in vitro* cell experiments and rat PCOS models. Further clinical trials are required to validate our findings and to evaluate the effectiveness and safety of NAC therapy in patients with PCOS. Second, the short duration of the intervention limits our understanding of the chronic effects of NAC on reproductive and metabolic functions. Future studies should conduct long-term investigations and explore the underlying mechanisms of action to validate and extend these findings. Third, in the animal experiments, some groups, including the NAC intervention (100 and 400 mg/kg body weight) control and PCOS groups, had relatively small sample sizes, which may have reduced the statistical power for comparisons of relevant parameters between these groups. Fourth, we did not measure ROS levels in HepG2 cells or serum levels of free testosterone, nor did we assess hepatic SHBG expression in rats by *in situ* hybridization. Measurement of these indicators would strengthen the interpretation of the findings.

## 5 Conclusion

This study indicates that NAC treatment can meliorate the oxidative stress-induced downregulation of SHBG and HNF4α in HepG2 cells and restore hepatic SHBG production by upregulating HNF4α and downregulating COUP-TF1/PPAR-γ expression in PCOS rat models. We also demonstrated that NAC at doses of 100, 200, and 400 mg/kg doses had different effects on hormones, glycolipid metabolism, and oxidative stress, with 200 mg/kg producing the most favorable overall effect in a rat model of PCOS. These findings indicate that NAC may be a potential complementary therapy for PCOS. Future PCOS management strategies should prioritize precise redox regulation using optimized NAC doses as a complementary approach alongside lifestyle interventions to reserve beneficial redox signaling while avoiding excessive antioxidant effects.

## Data Availability

All data and materials supporting the conclusions of this study are included within the article and supplementary data. Further inquiries can contact the corresponding author.
