# Supplementary material for: N-acetylcysteine ameliorates oxidative stress-induced decline in sex hormone-binding globulin in HepG2 cells and polycystic ovary syndrome rats
Source: Front Pharmacol. 2026 Oct 13;17:1930645. doi: 10.3389/fphar.2026.1930645 (PMC13710105; doi:10.3389/fphar.2026.1930645)

The original images of Western Blot in HepG2 cells

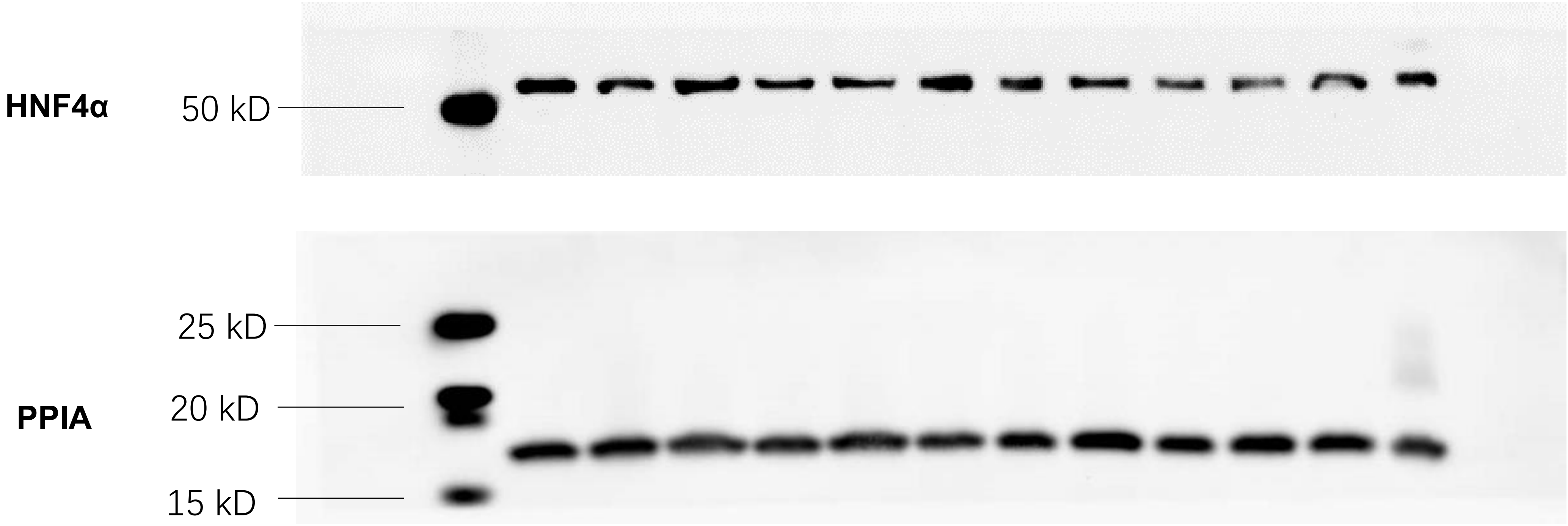

The original images of Western Blot in HepG2 cells

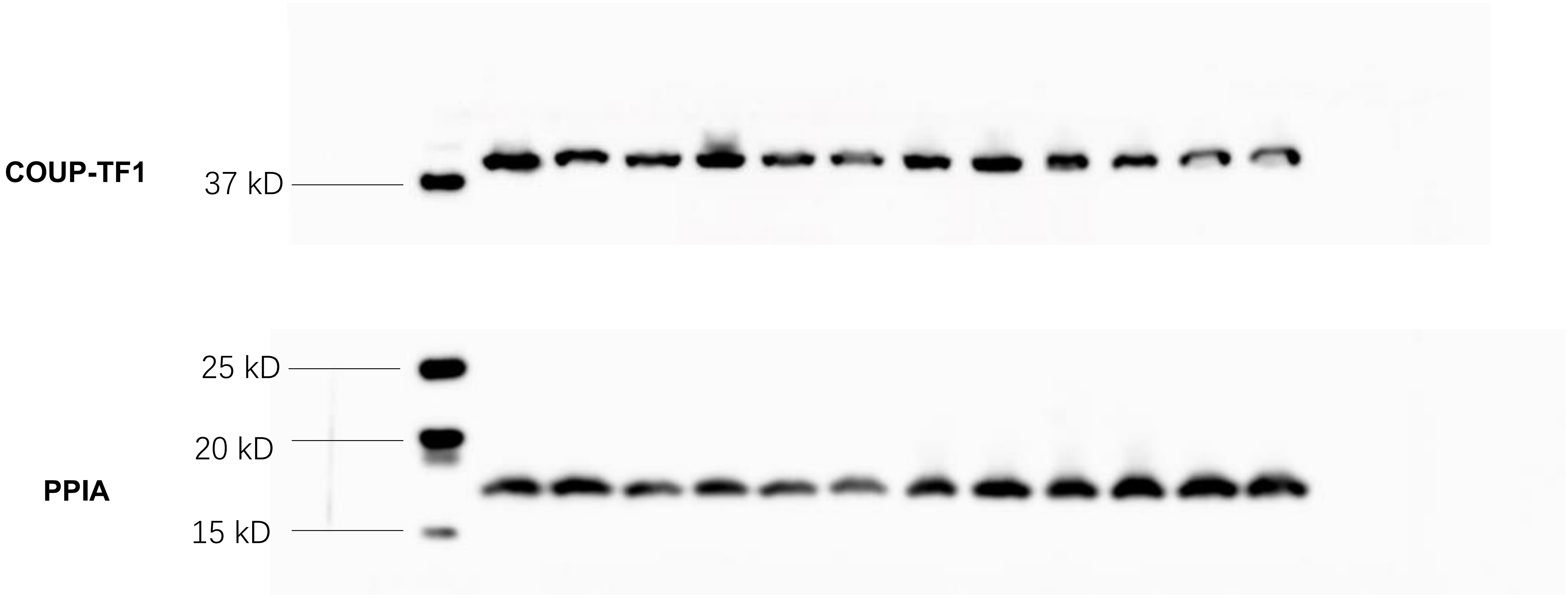

The original images of Western Blot in HepG2 cells

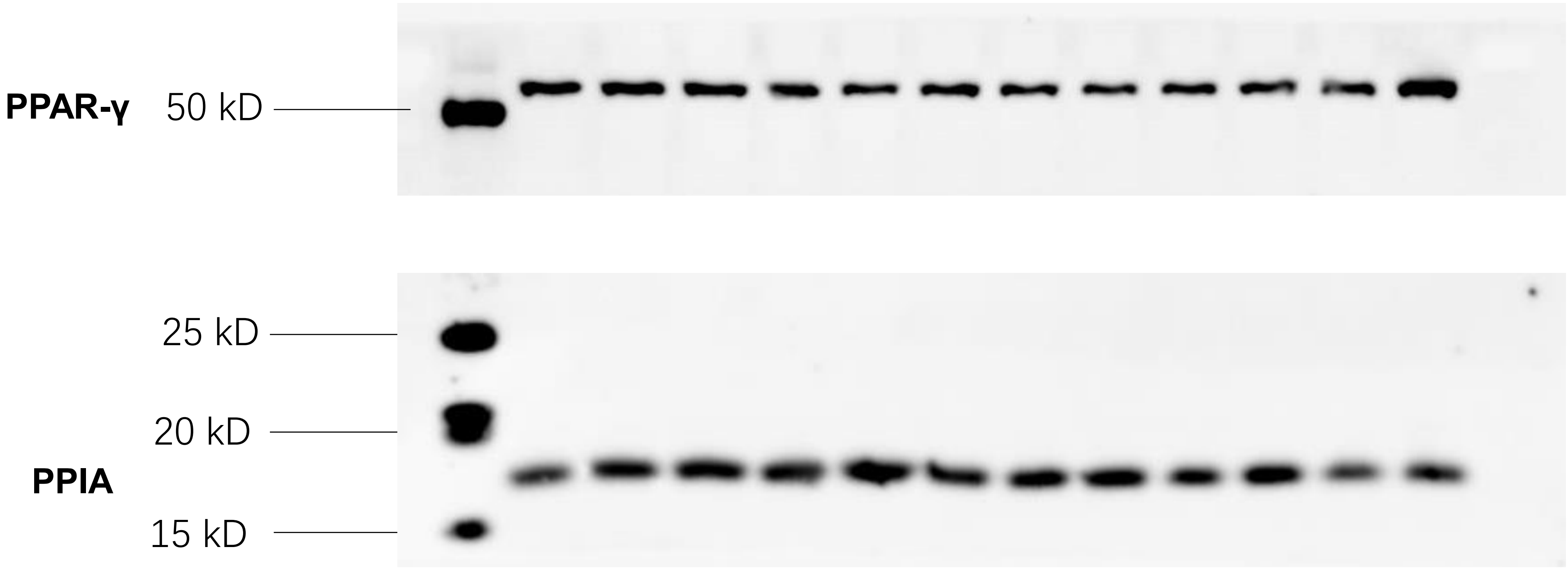

The original images of Western Blot in HepG2 cells

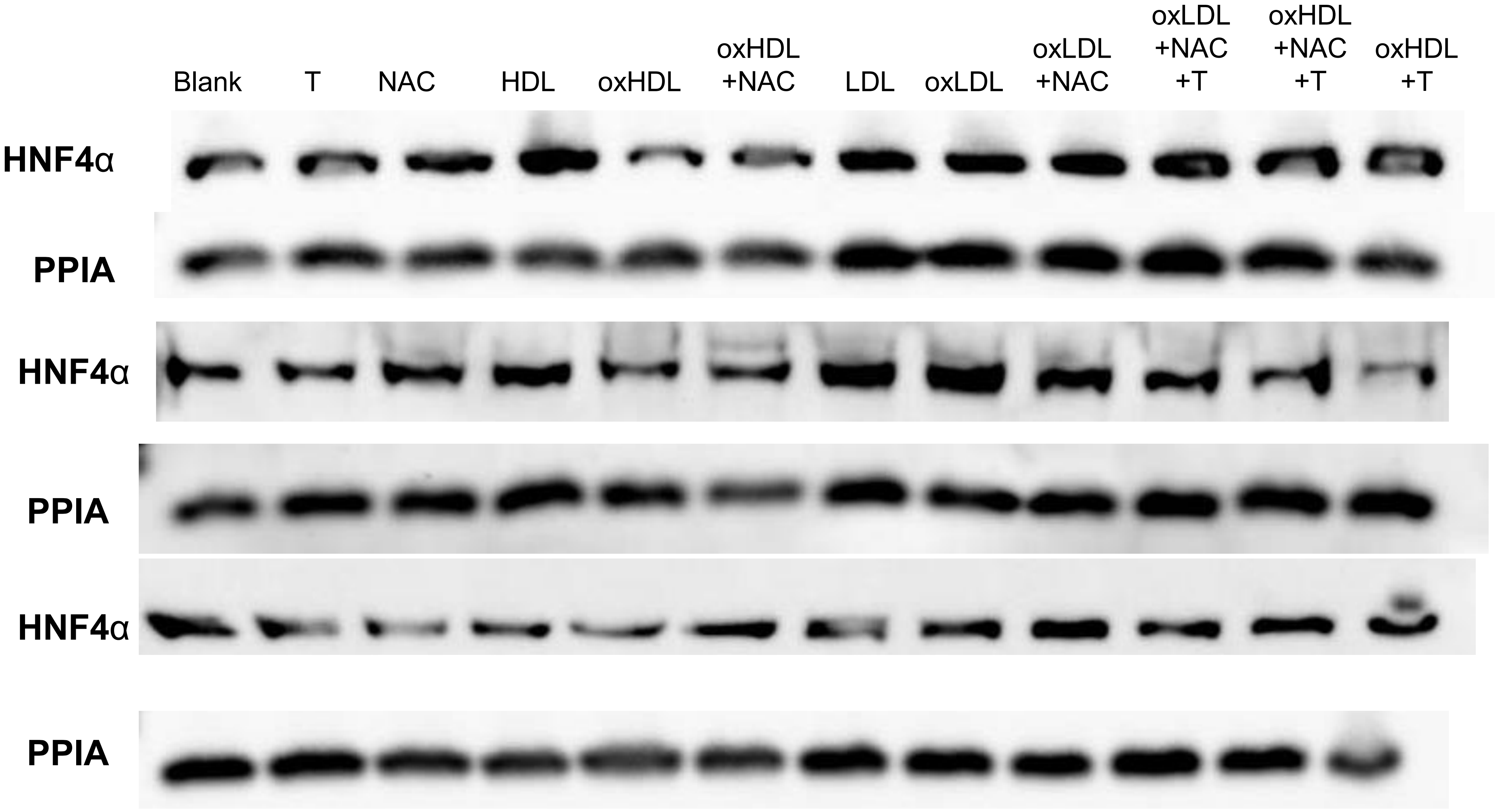

The original images of Western Blot in HepG2 cells

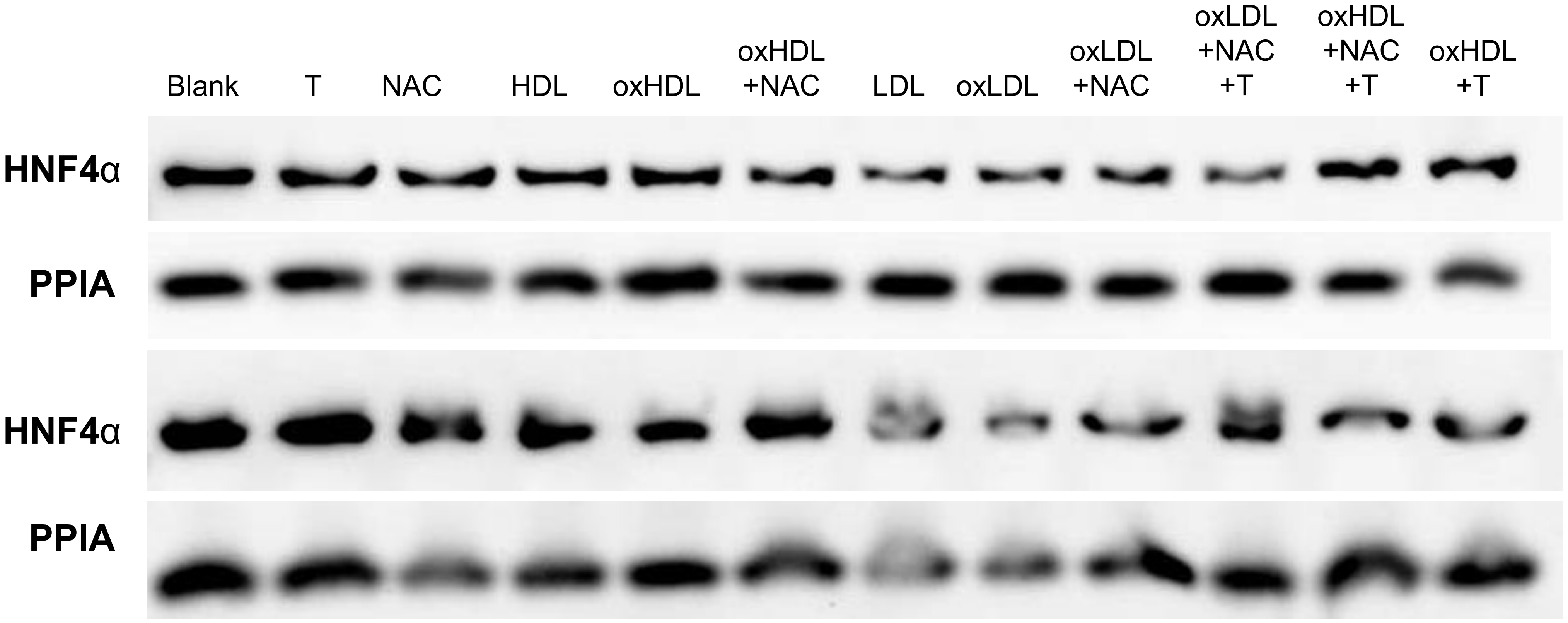

The original images of Western Blot in HepG2 cells

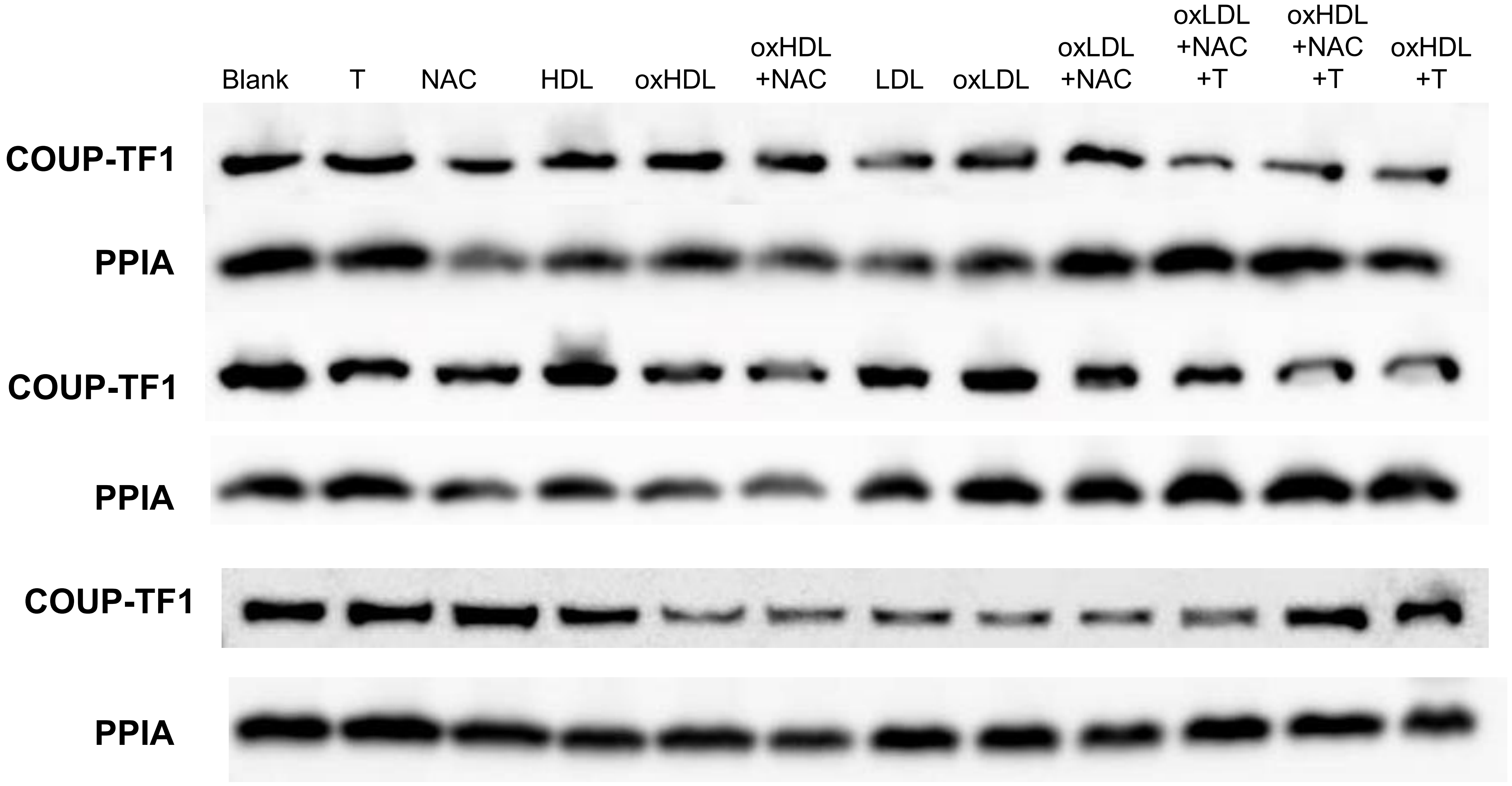

The original images of Western Blot in HepG2 cells

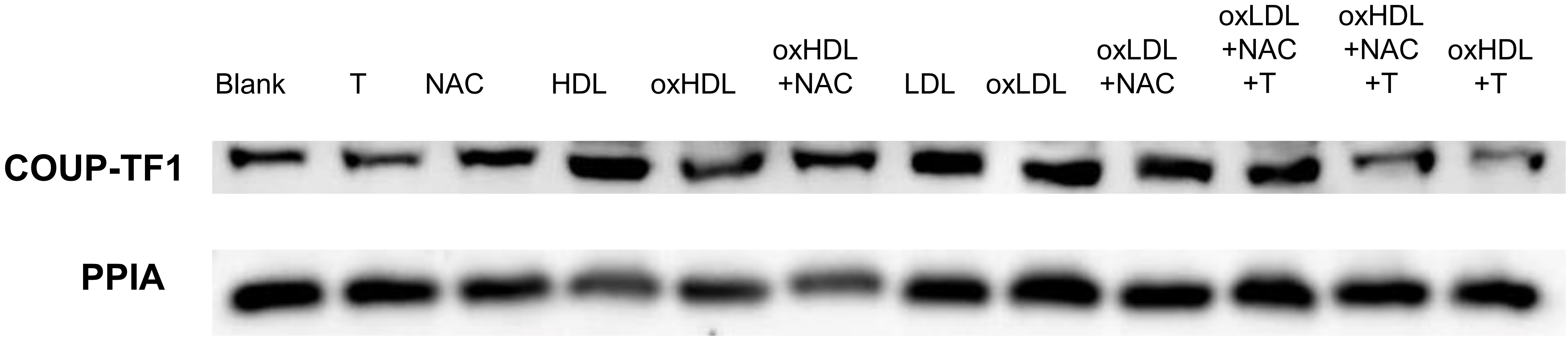

The original images of Western Blot in HepG2 cells

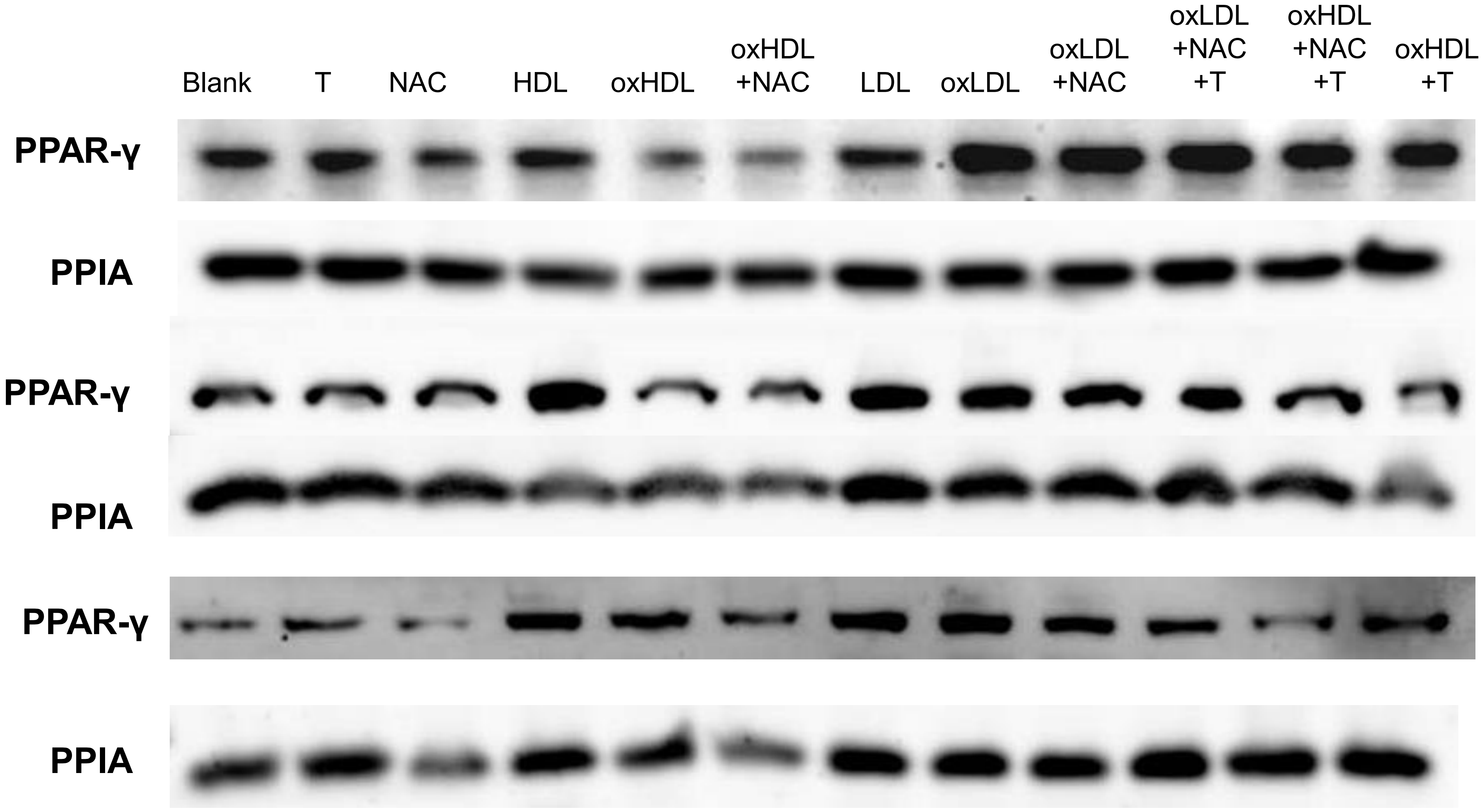

The original images of Western Blot in HepG2 cells

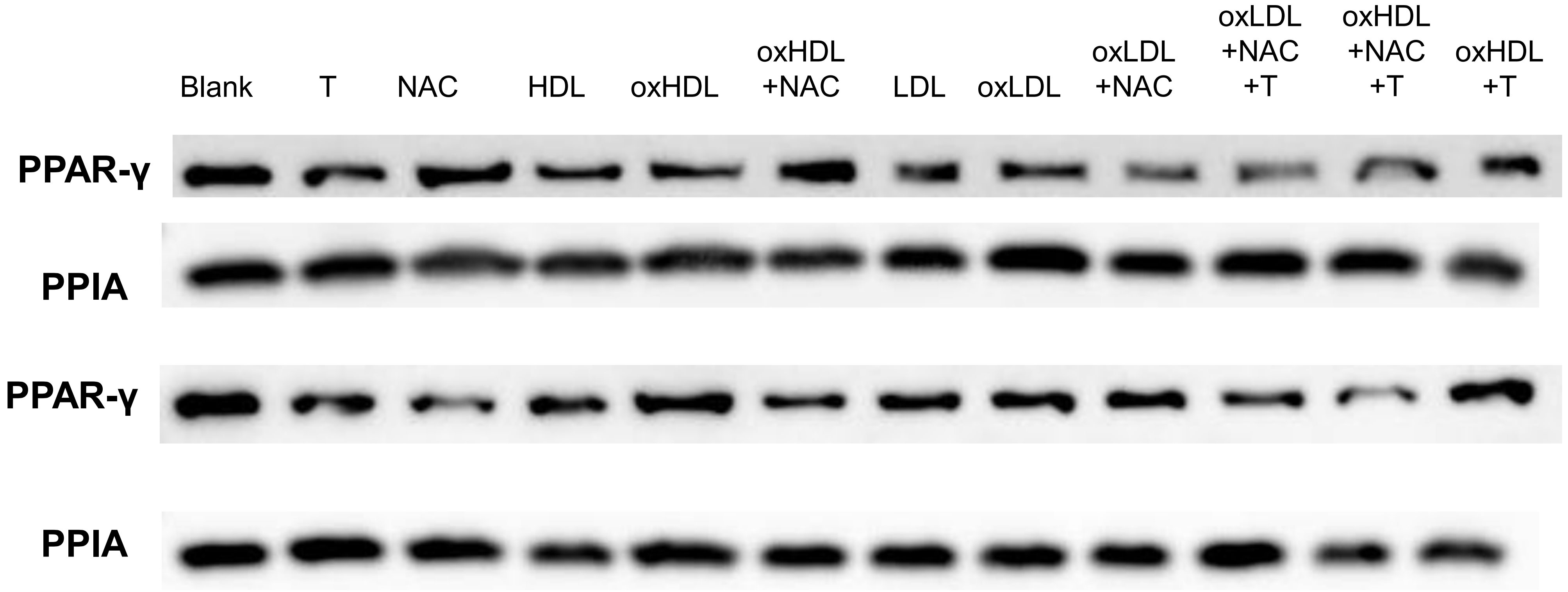

The original images of Western Blot in rat liver

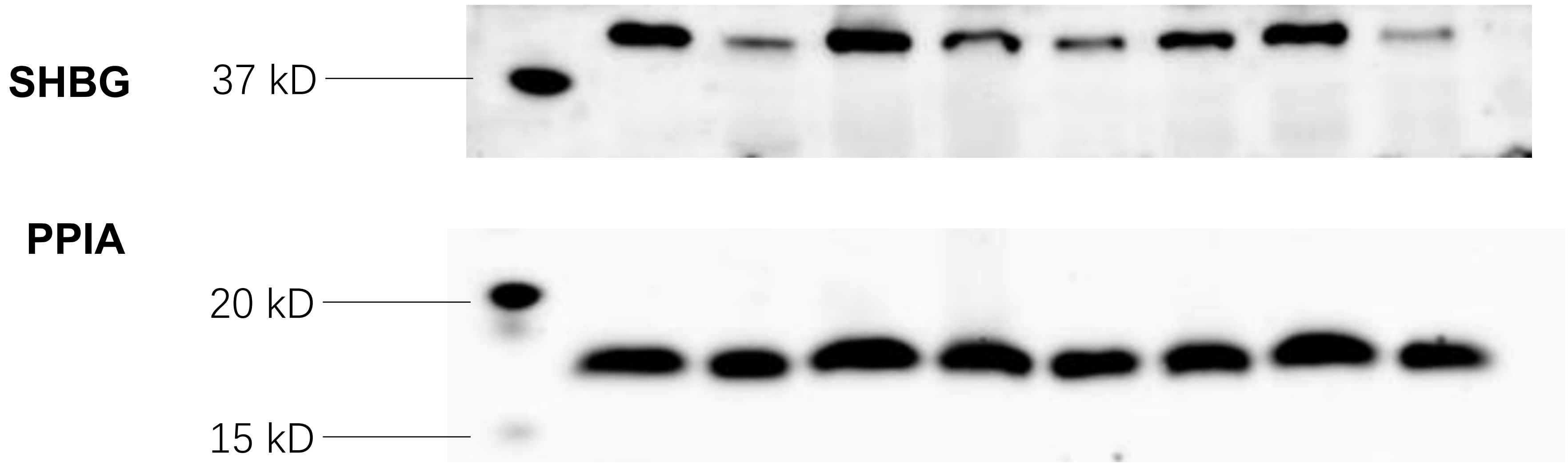

The original images of Western Blot in rat liver

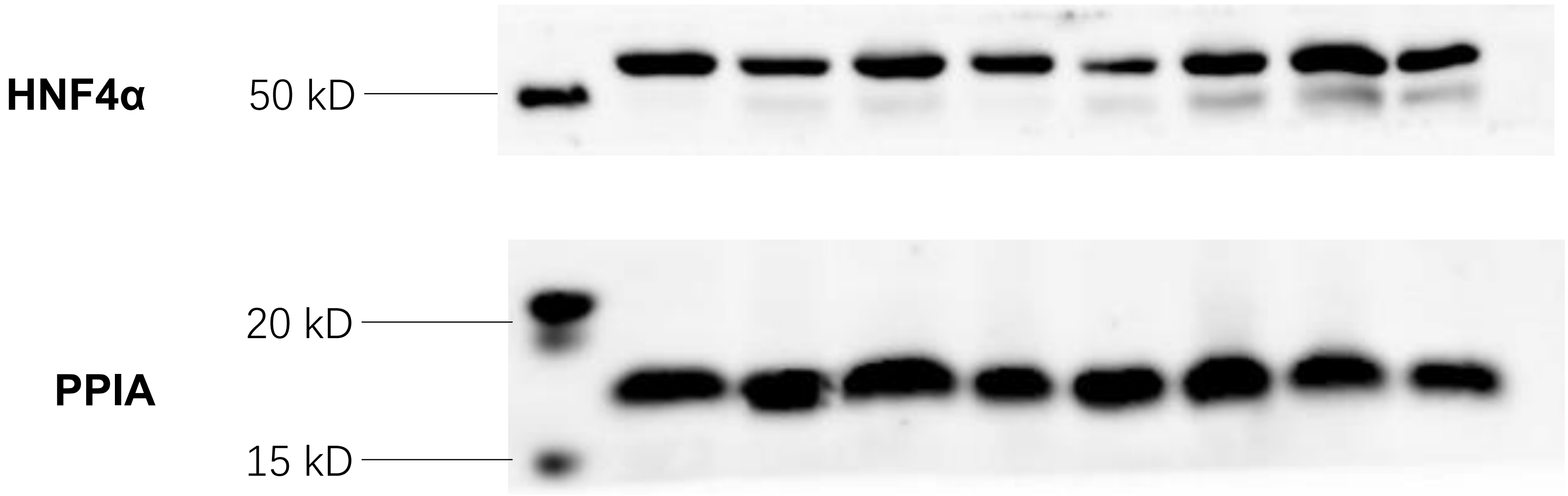

The original images of Western Blot in rat liver

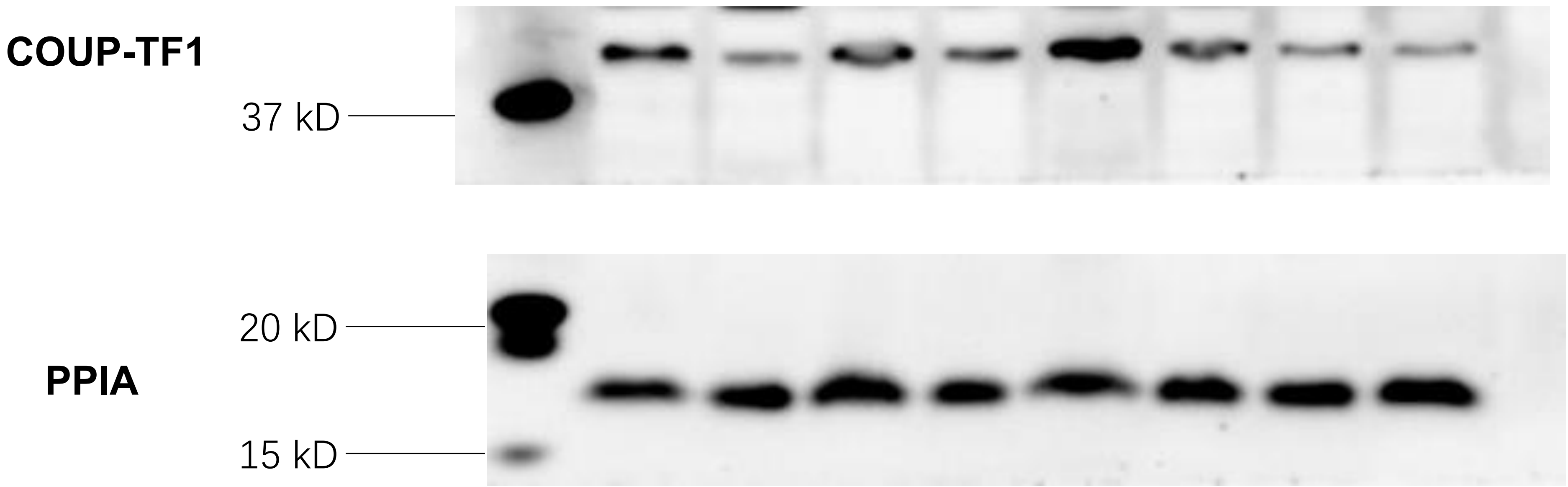

The original images of Western Blot in rat liver

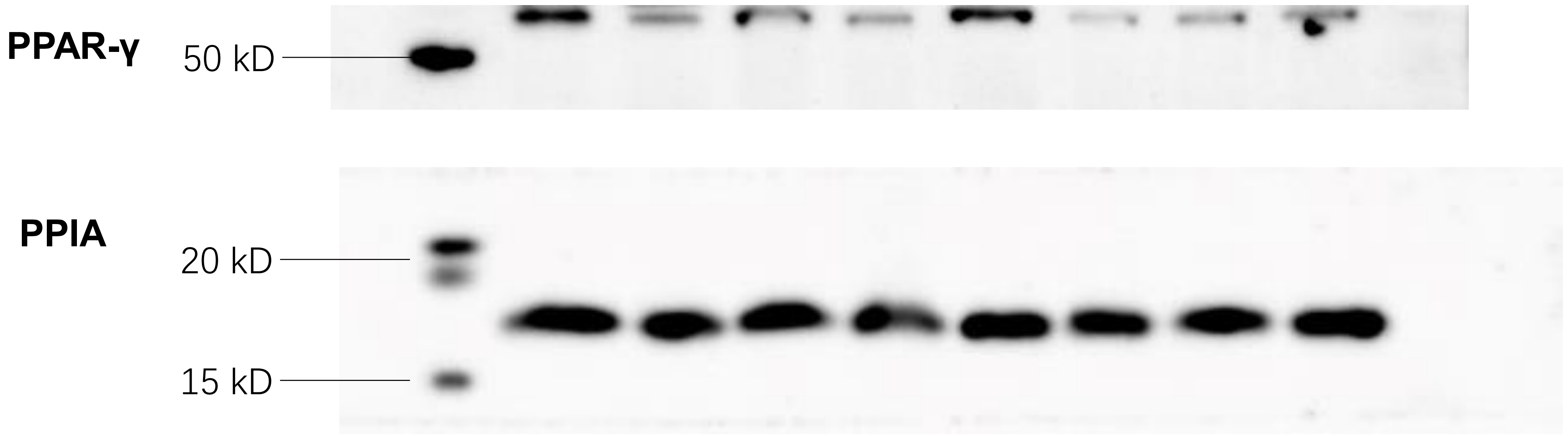

20231226

The original images of Western Blot in rat liver

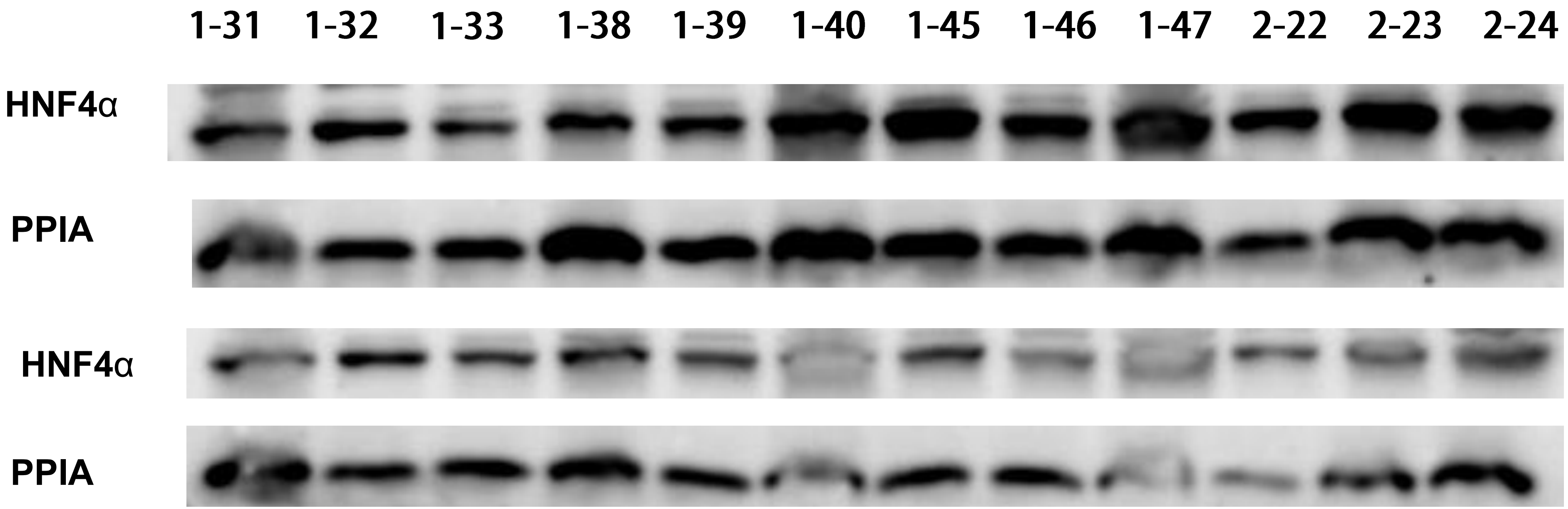

20231226

The original images of Western Blot in rat liver

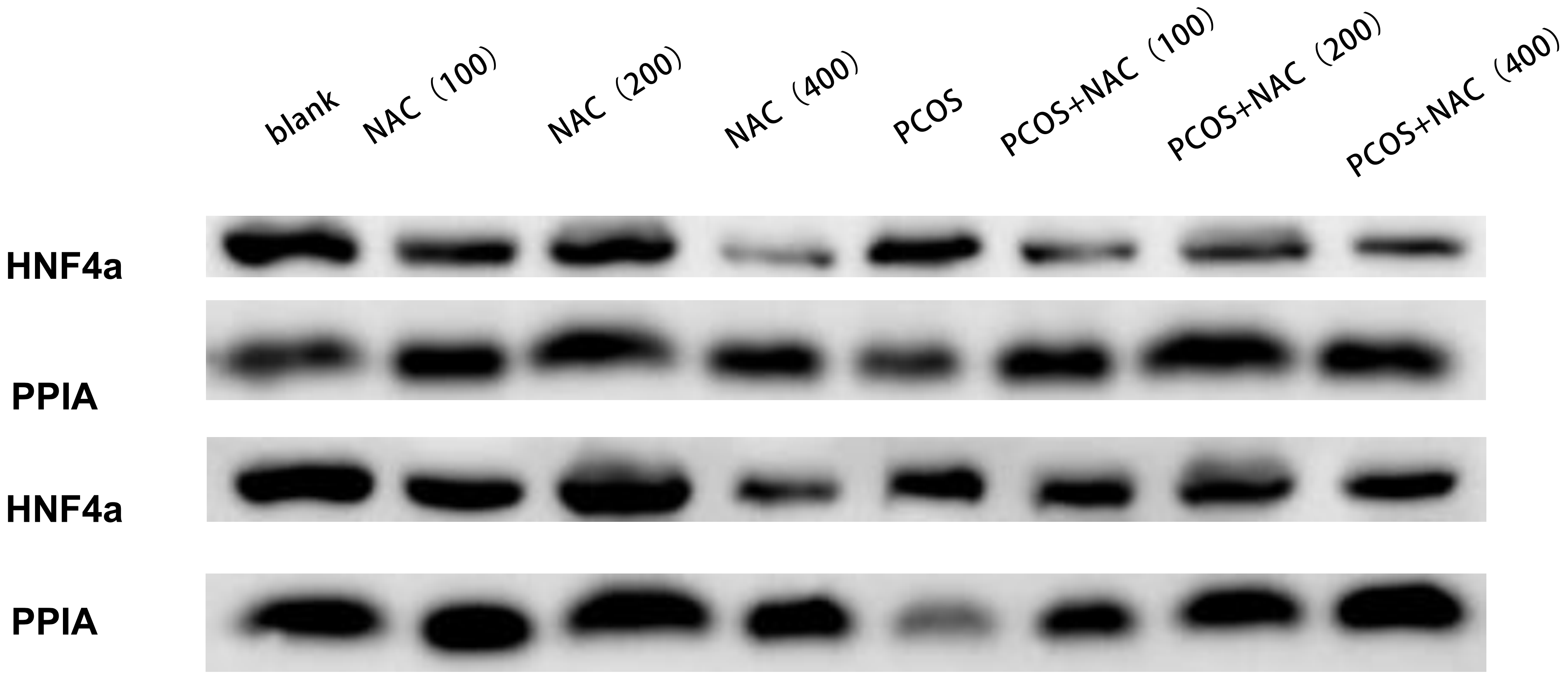

20240103

The original images of Western Blot in rat liver

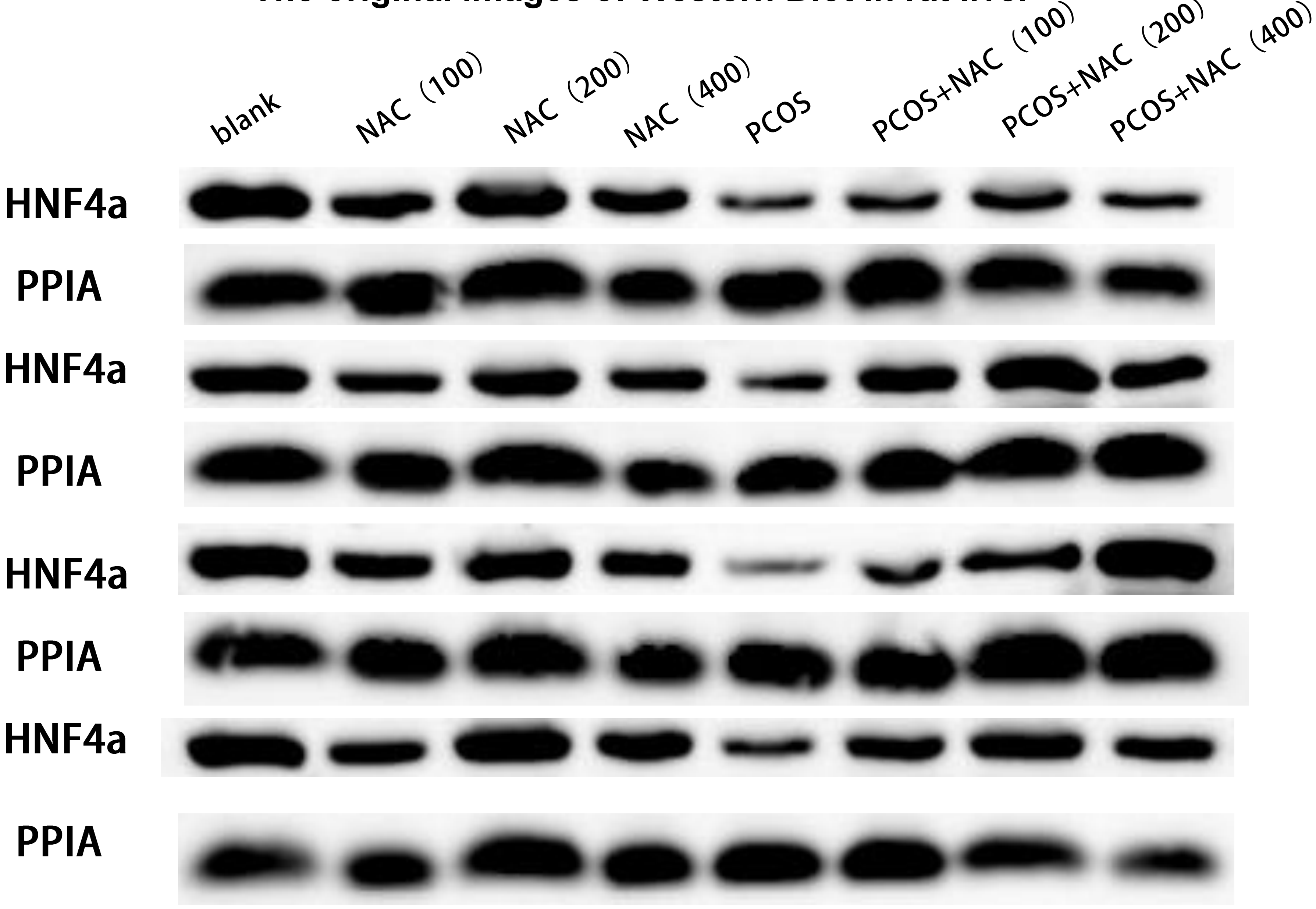

20240105

The original images of Western Blot in rat liver

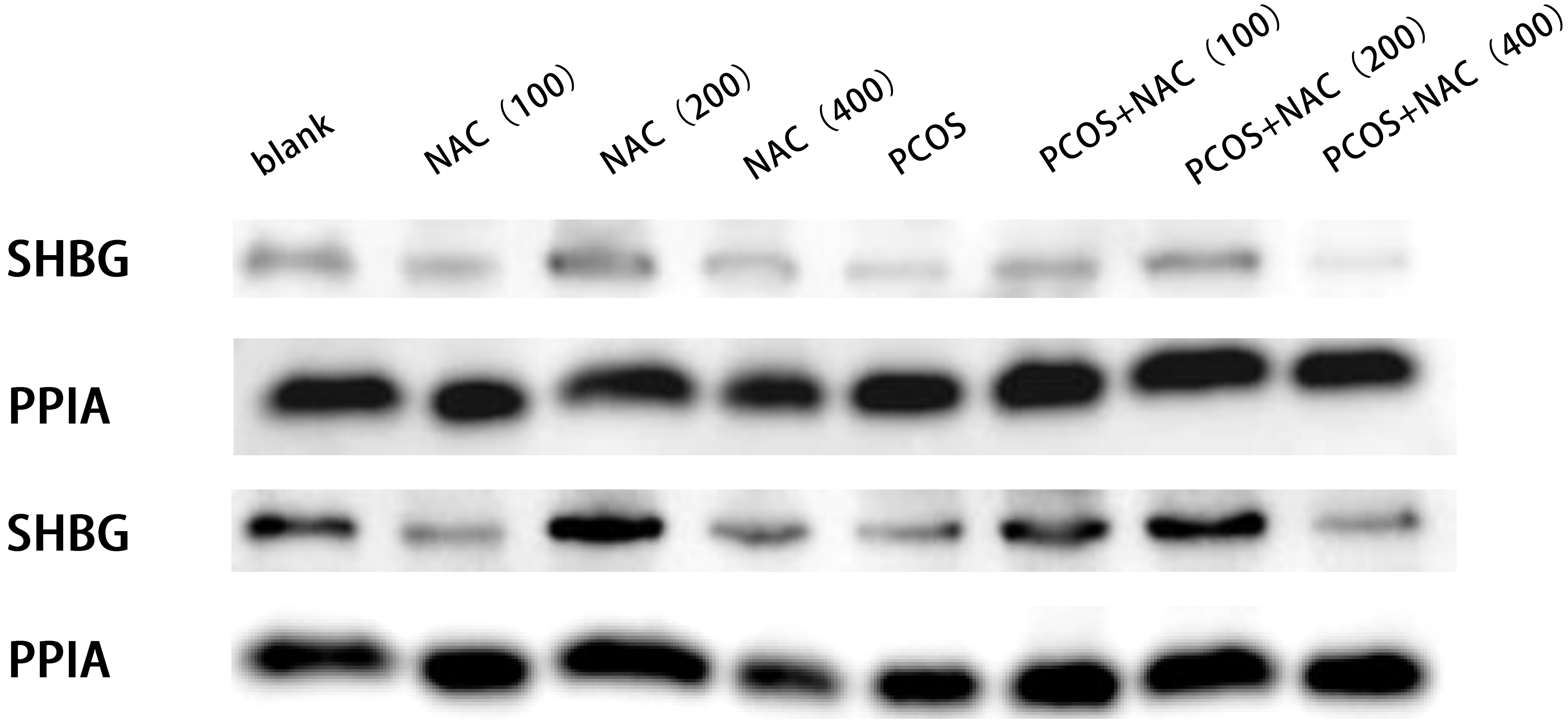

20240109

The original images of Western Blot in rat liver

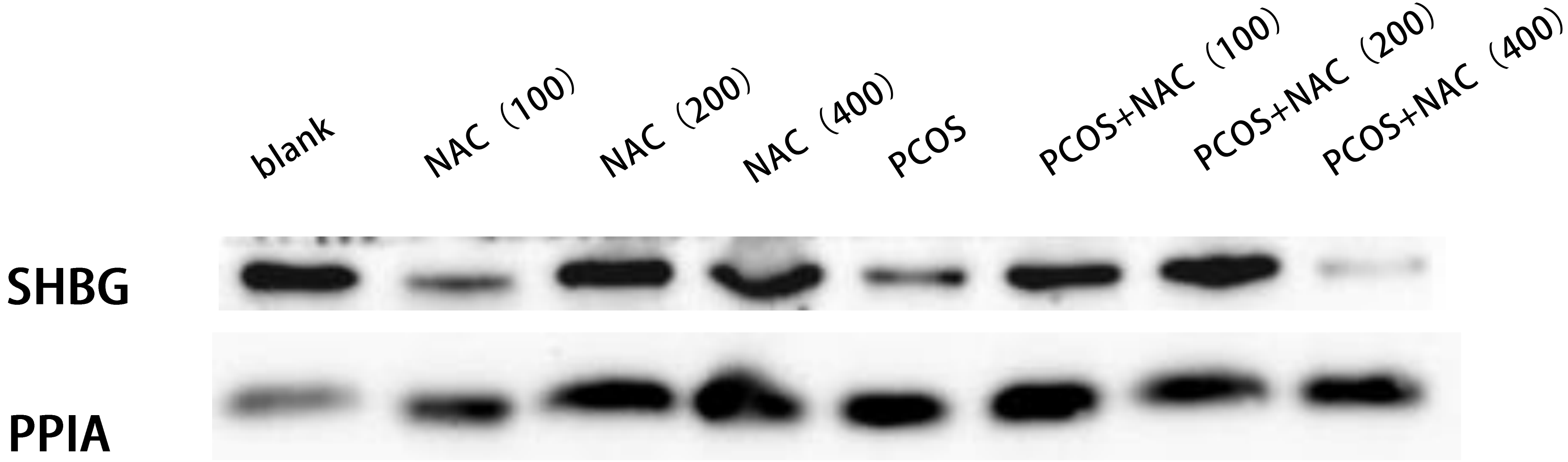

20240109

The original images of Western Blot in rat liver

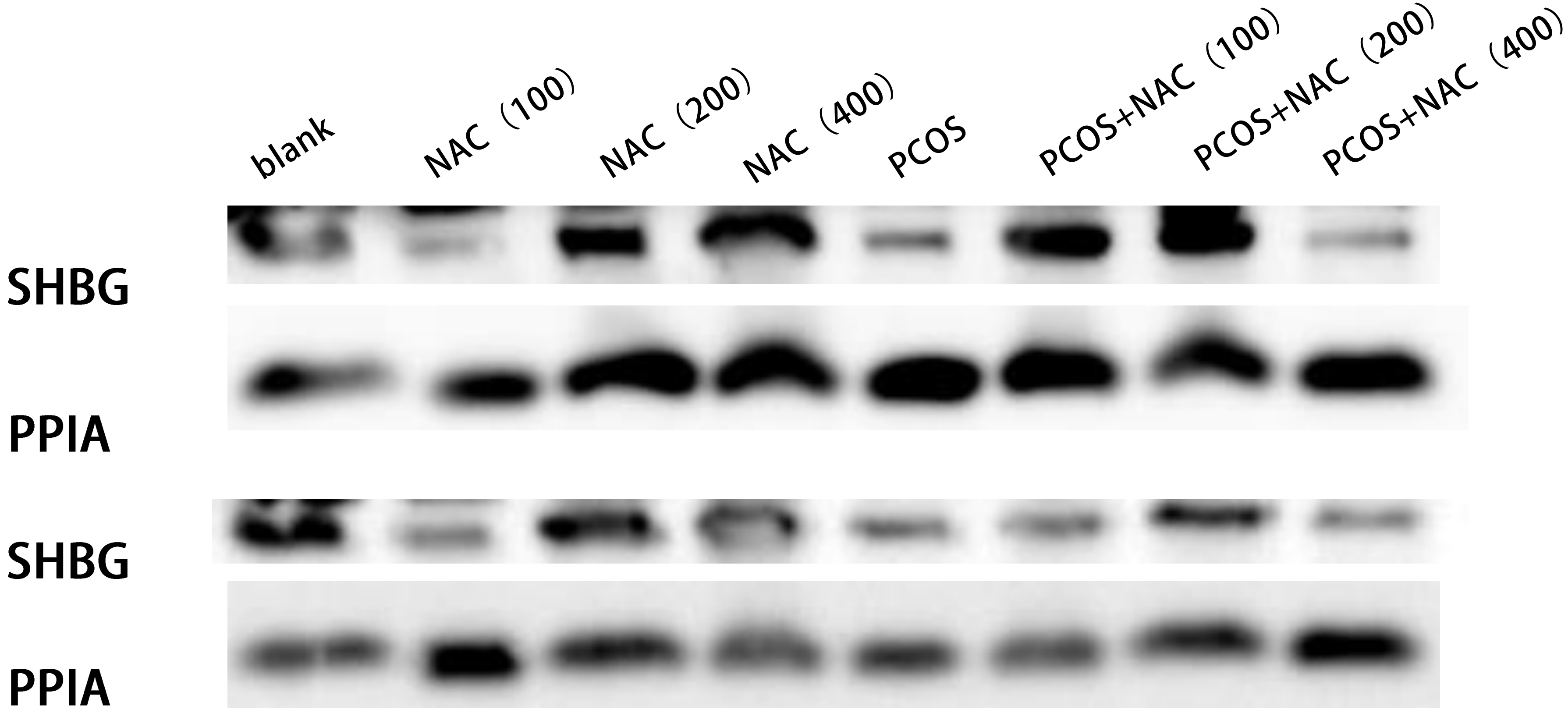

20240116

The original images of Western Blot in rat liver

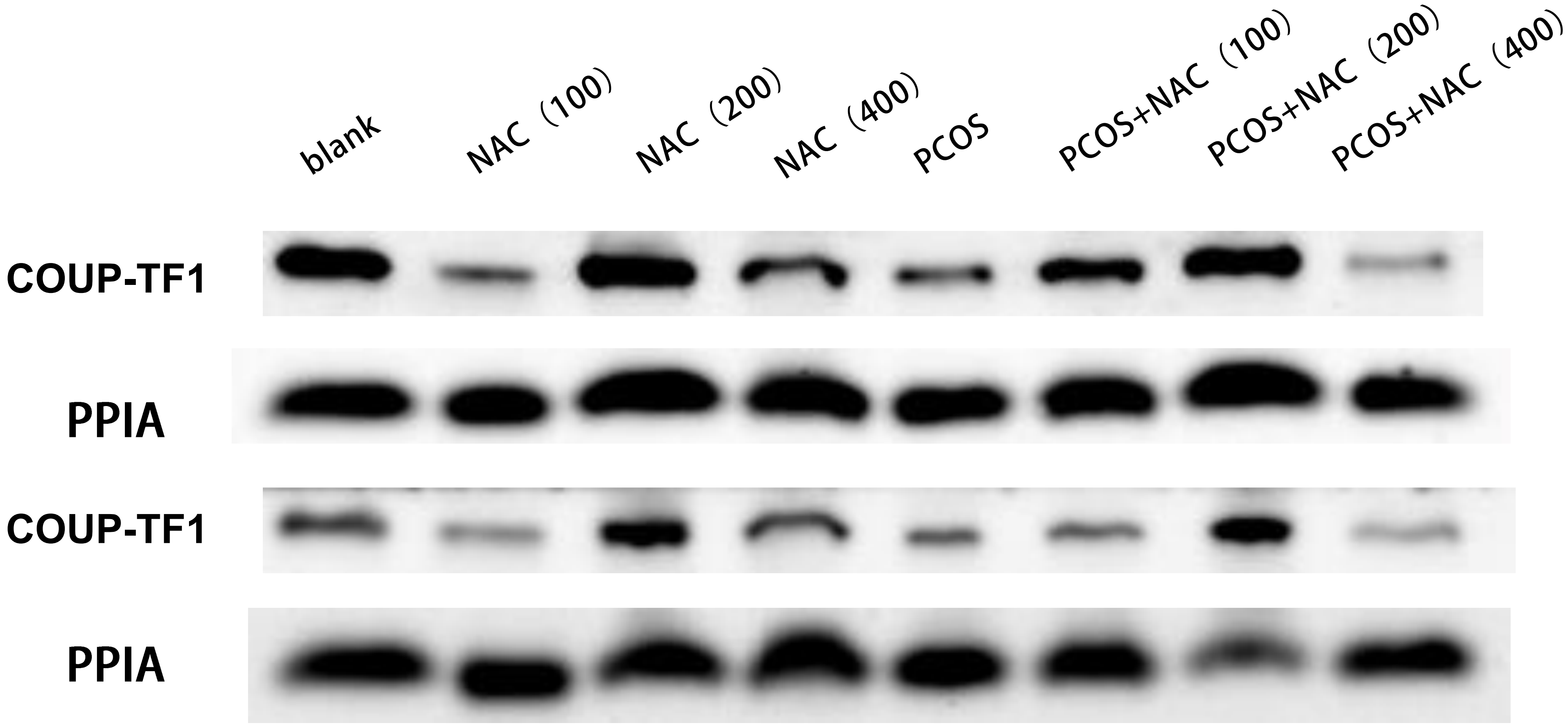

20240116 **The original images of Western Blot in rat liver**

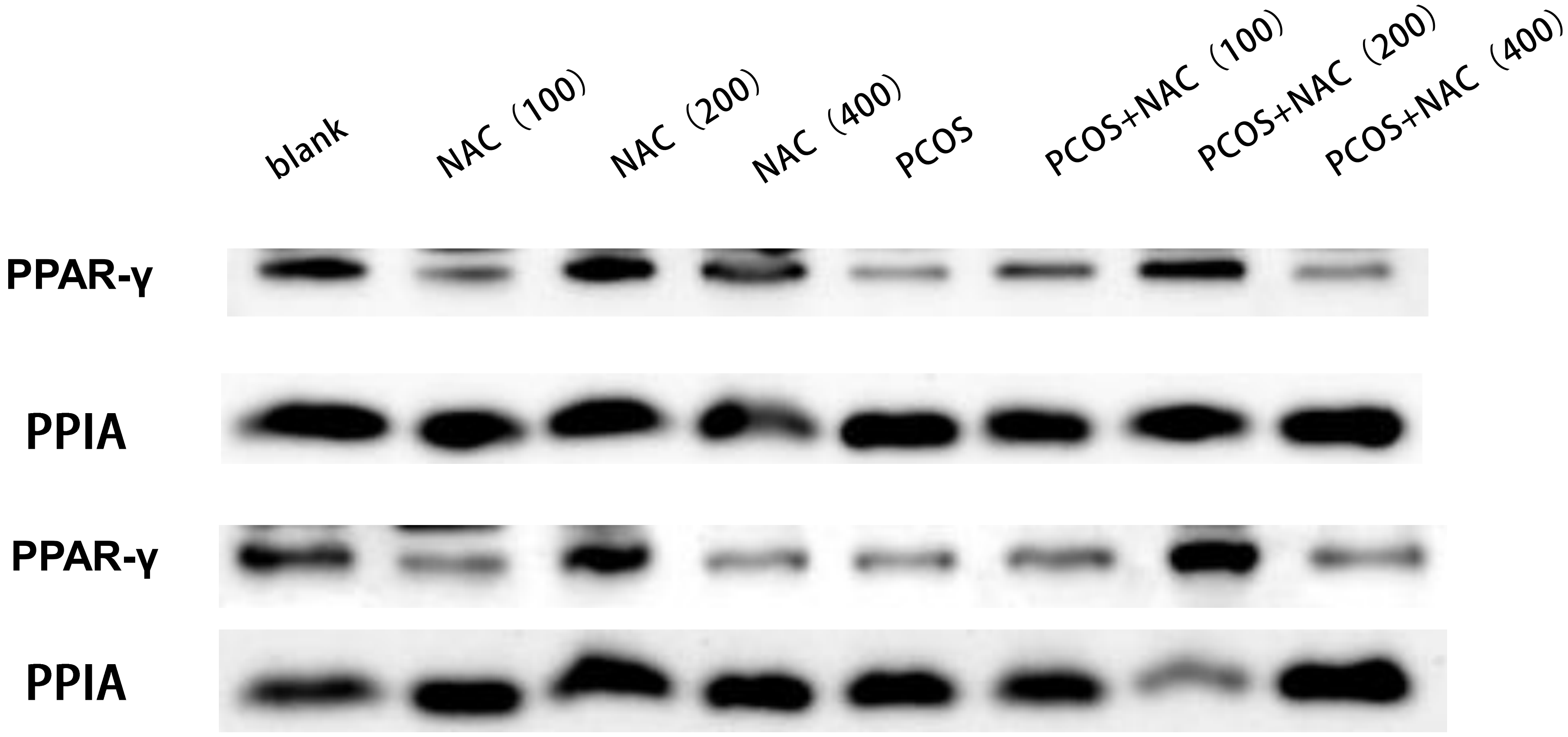

20240120

The original images of Western Blot in rat liver

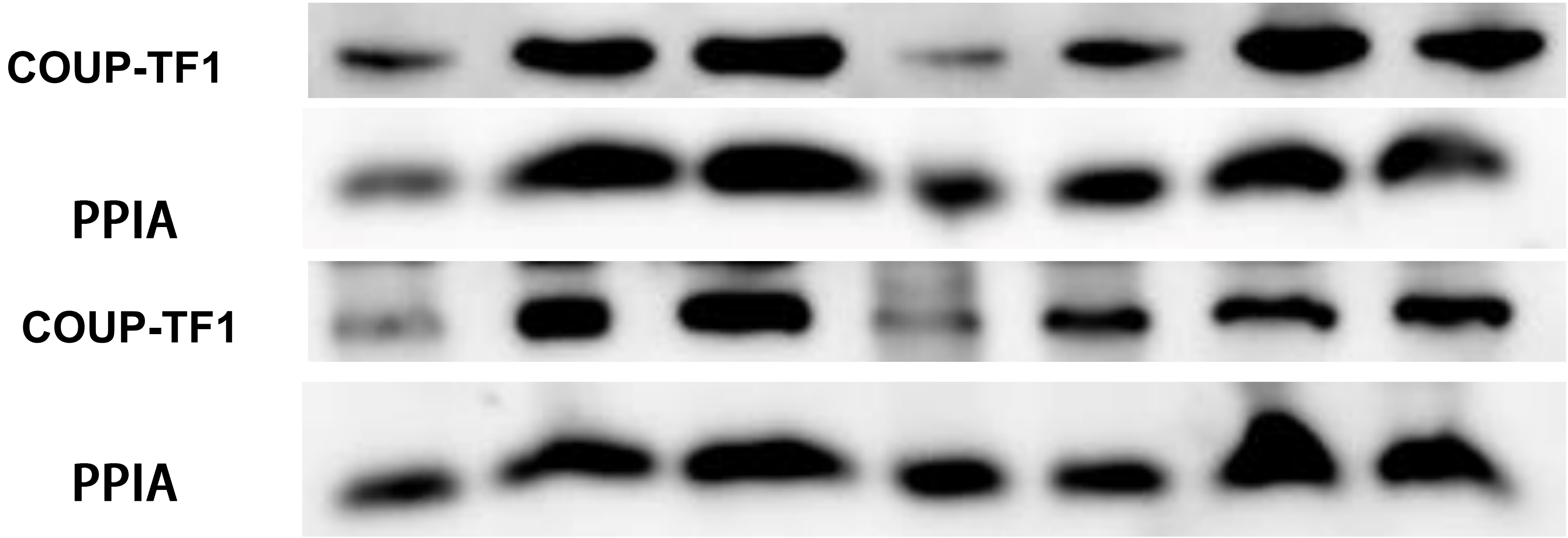

20240120

The original images of Western Blot in rat liver

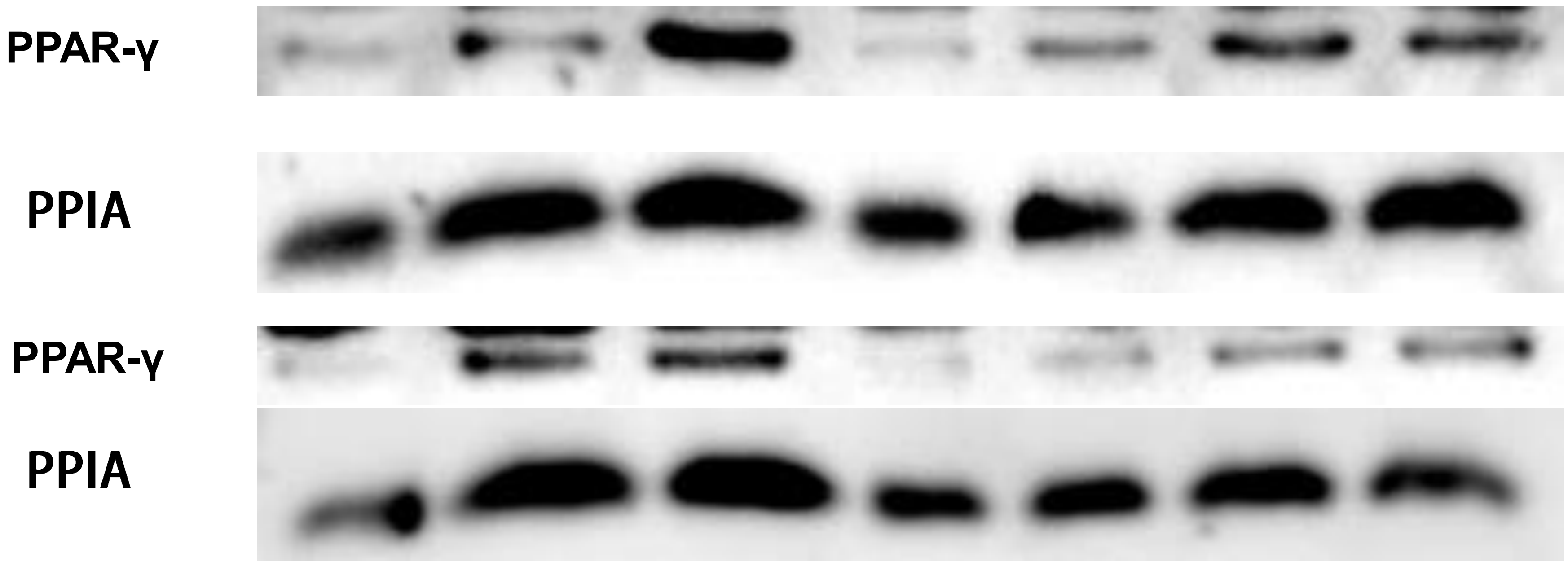

20240124

The original images of Western Blot in rat liver

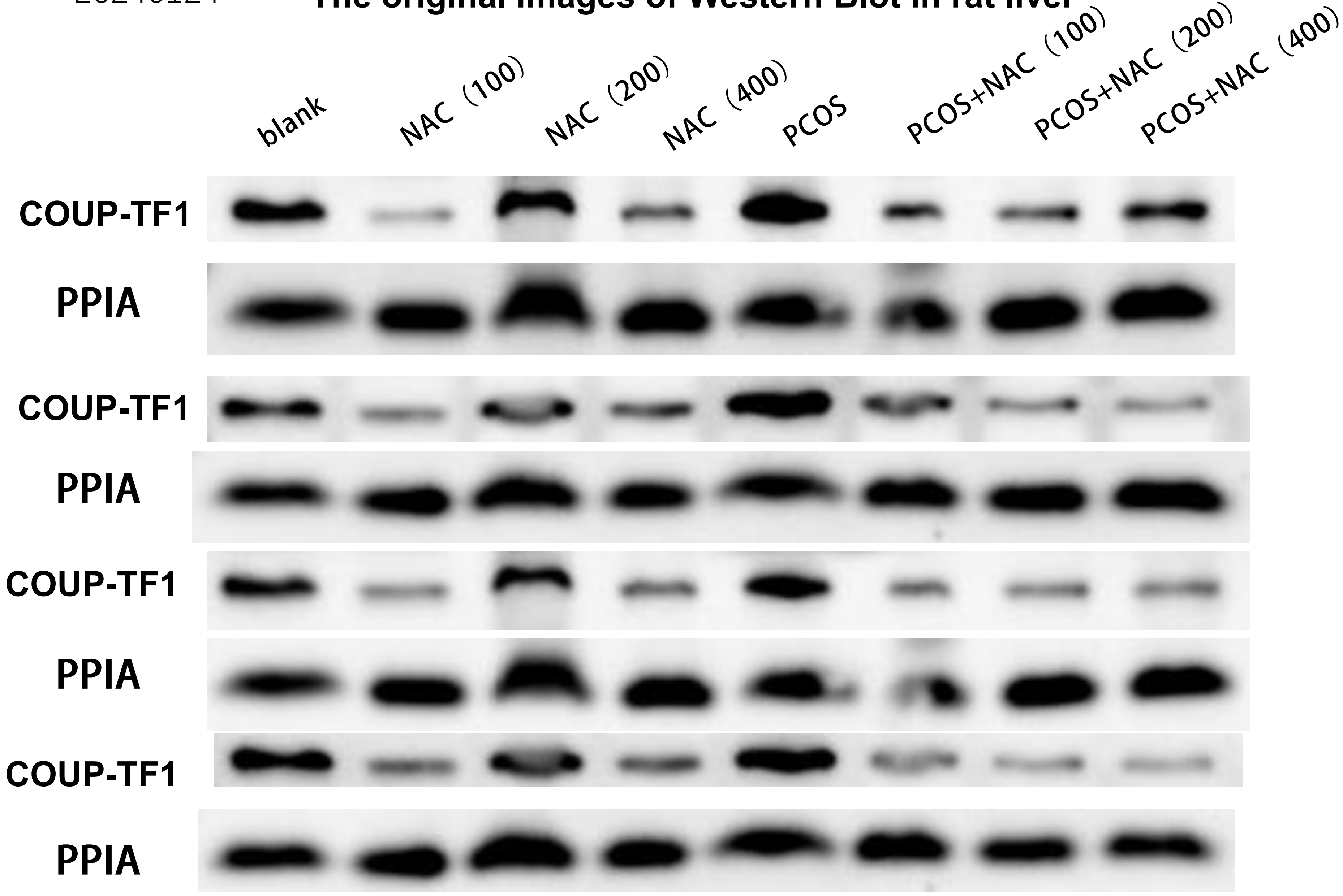

20240124

The original images of Western Blot in rat liver

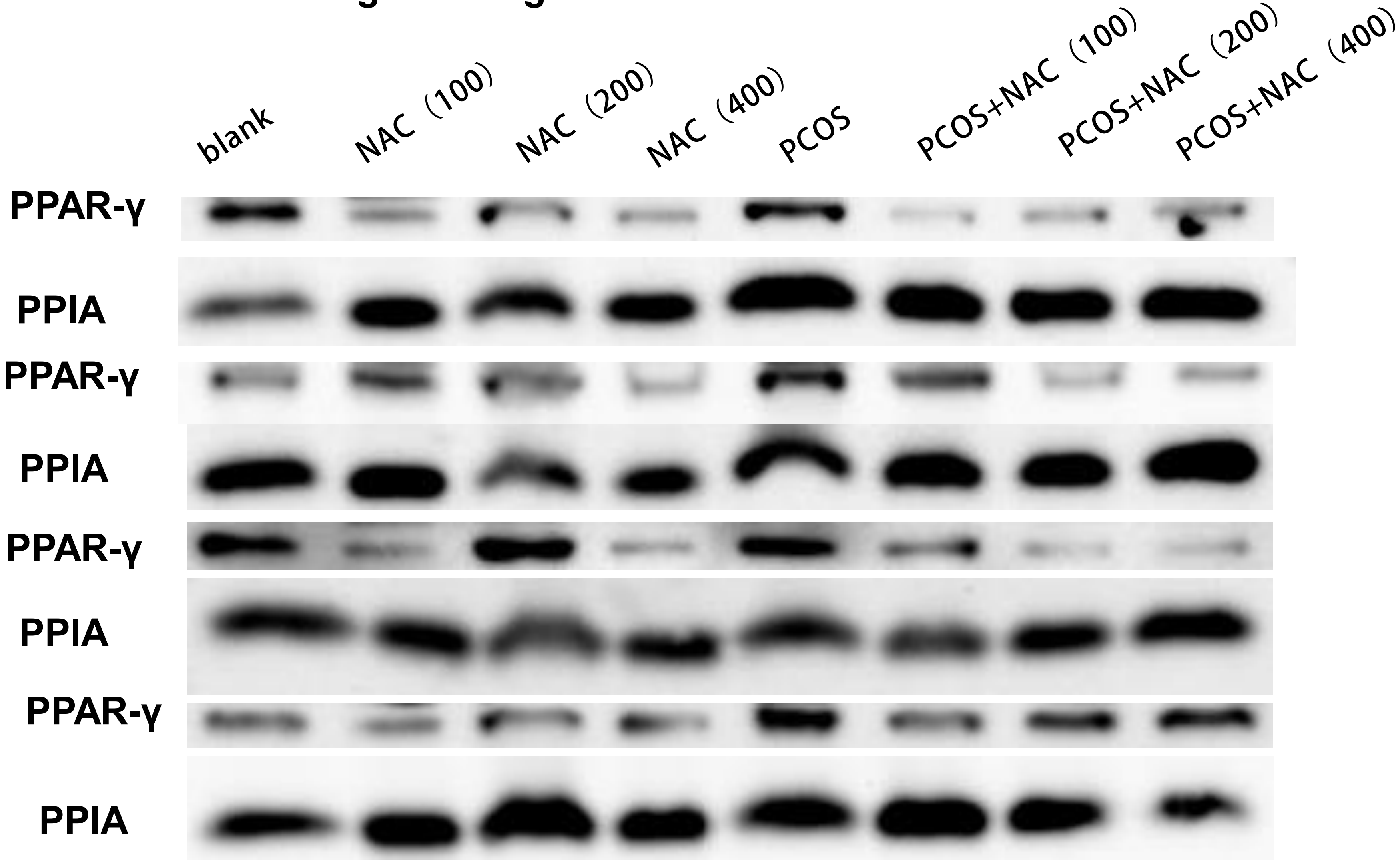

Supplement: Supplementary file 1 [file DataSheet1.pdf]
