# Supplementary material for: N-acetylcysteine ameliorates oxidative stress-induced decline in sex hormone-binding globulin in HepG2 cells and polycystic ovary syndrome rats
Source: Front Pharmacol. 2026 Oct 13;17:1930645. doi: 10.3389/fphar.2026.1930645 (PMC13710105; doi:10.3389/fphar.2026.1930645)
Supplement: Supplementary file 2 [file Table1.docx]

**Supplemental Table 1. Sequences of primer pairs used for qRT-PCR measurement**

| Gene |  | Primer Sequence (5**′**-3**′**) | Product Size (bp) | |
| --- | --- | --- | --- | --- |
| Human *HNF4*α **^[1]^** | Forward | TGCGACTCTCCAAAACCCTC | 128 | |
|  | Reverse | TGATGGGGACGTGTCATTGC |  | |
| Human *SHBG* **^[2]^** | Forward | GCACCAGGTGGAAGTCAAGAT | 233 | |
|  | Reverse | AGATCTCGGCCTGTTTGTCC |  | |
| Human *COUP-TF1* | Forward | CTTACACATGCCGTGCCAAC | 132 | |
| (*NR2F1*) **^[2]^** | Reverse | GAGGCATTCTTCCTCGCTGA |  | |
| Human *PPAR-γ* **^[3]^** | Forward | TGAAGCTGAACCACCCTGAGT | 90 | |
|  | Reverse | GACCGTGTTCCGTGACAATC |  | |
| Human *18S* **^[4]^** | Forward | TAACGAACGAGACTCTGGCAT | 138 | |
|  | Reverse | CGGACATCTAAGGGCATCACAG |  | |
| Rat *Hnf4α* | Forward | CCATGGGCAATGACACATCC | 174 | |
|  | Reverse | TGTGGTTCTTCCTCACGCTC |  | |
| Rat *Shbg* | Forward | GACGGACCCTGAGACACATTG | 137 | |
|  | Reverse | GGAGGAAGAGGGTTTGCTGAT |  | |
| Rat *Coup-tf1* | Forward | GCGGCAAGCACTACGGC | 95 | |
| *(Nr2f1)* | Reverse | GTTGGCACGGCATGTGTAA |  | |
| Rat *Ppar-γ* | Forward | ATCCTCCTGTTGACCCAGAGC | 152 | |
|  | Reverse | GGAGTGGTCATCCATCACAGA |  | |
| Rat *18s* | Forward | CCGGCTTTCCCAAAATGCTC | 126 | |
|  | Reverse | GGTGTCTGGTCCTTTCTCGG |  |  |

*HNF4α* (*Hnf4α*), hepatocyte nuclear factor 4α; *COUP-TF1* (*Coup-tf1*), chicken ovalbumin upstream promoter-transcription factor 1; PPAR-γ (*Ppar-γ*), peroxisome proliferator-activated receptor-γ; SHBG (Shbg), sex hormone-binding globulin.

The real-time PCR primers for the rat *Shbg*, *Hnf4α*, *Coup-tf1,* *Ppar-γ,* and *18s* genes were designed using Primer-BLAST.

**References**

[1] Khole S, Mittal S, Jagadish N, Ghosh D, Gadgil V, Sinkar V and Ghaskadbi S. Andrographolide enhances redox status of liver cells by regulating microRNA expression. Free Radic Biol Med. 2019;130:397-407. <https://doi.org/10.1016/j.freeradbiomed.2018.11.004>.

[2] Sun Y, Li S, Liu H, Bai H, Hu K, Zhang R, Liu Q and Fan P. Oxidative stress promotes hyperandrogenism by reducing sex hormone-binding globulin in polycystic ovary syndrome. Fertil Steril. 2021;116:1641-50. <https://doi.org/10.1016/j.fertnstert.2021.07.1203>.

[3] Saez-Lopez C, Soriguer F, Hernandez C, Rojo-Martinez G, Rubio-Martin E, Simo R, et al. Oleic acid increases hepatic sex hormone binding globulin production in men. Mol Nutr Food Res, 2014, 58:760-7. <https://doi.org/10.1002/mnfr.201300304>.

[4] Simo R, Barbosa-Desongles A, Lecube A, Hernandez C, Selva DM. Potential Role of Tumor Necrosis Factor-α in Downregulating Sex Hormone–Binding Globulin. Diabetes, 2012, 61:372-82. <https://doi.org/10.2337/db11-0727>.
