# Supplementary material for: N-acetylcysteine ameliorates oxidative stress-induced decline in sex hormone-binding globulin in HepG2 cells and polycystic ovary syndrome rats
Source: Front Pharmacol. 2026 Oct 13;17:1930645. doi: 10.3389/fphar.2026.1930645 (PMC13710105; doi:10.3389/fphar.2026.1930645)
Supplement: Supplementary file 3 [file Table2.docx]

**Supplementary Table 2. Primary antibodies for western blot: sources, species, catalog number, predicted band size, dilution, and company**

| Antibody | Sources | Species | Cat.No. | kDa | Dilution | Company |
| --- | --- | --- | --- | --- | --- | --- |
| HNF4α | Rabbit | Human | 3113 | 52 | 1:1000 | CST |
| COUP-TF1 | Rabbit | Human, Rat | 6364 | 46 | 1:1000 | CST |
| PPAR-γ | Rabbit | Human | 2435 | 57 | 1:1000 | CST |
| PPIA | Rabbit | Human, Rat | 2175 | 18 | 1:1000 | CST |
| HNF4α | Rabbit | Rat | 201460 | 53 | 1:1000 | Abcam |
| PPAR-γ | Rabbit | Rat | 272718 | 57 | 1:1000 | Abcam |
| SHBG | Rabbit | Rat (expected) | 126617 | 44 | 1:1000 | Abcam |

HNF4α, hepatocyte nuclear factor 4α; COUP-TF1, chicken ovalbumin upstream promoter-transcription factor 1; PPAR-γ, peroxisome proliferator-activated receptor-γ; PPIA, peptidylprolyl isomerase A; SHBG, sex hormone-binding globulin. CST (Cell Signaling Technology, Danvers, MA); Abcam (Abcam Shanghai Trading Co., Ltd., Shanghai, China)
